# Continuous Inhibition-Zone Modeling and Binary Classification for *Pseudomonas aeruginosa* Hit Prioritization: A Retrospective QSAR Evaluation

**DOI:** 10.3390/ph19081173

**Published:** 2026-07-27

**Authors:** Sukrit Kashyap, Barlina Konwar, Ji Young Lee, Kwang-sun Kim

**Affiliations:** Department of Chemistry and Chemistry Institute for Functional Materials, Pusan National University, Busan 46241, Republic of Korea; sukrit.kshp@gmail.com (S.K.); barlinakonwar100@gmail.com (B.K.); ljycielo@gmail.com (J.Y.L.)

**Keywords:** *Pseudomonas aeruginosa*, antibacterial QSAR, inhibition-zone modeling, drug discovery, continuous endpoint modeling, hit prioritization, machine learning

## Abstract

**Background/Objectives:** Antibiotic-resistant *Pseudomonas aeruginosa* and limited experimental validation capacity motivate efficient prioritization of antibacterial candidates from large chemical libraries. Quantitative structure–activity relationship (QSAR) benchmarks often binarize disk diffusion inhibition-zone (IZ) measurements, obscuring activity gradients and imposing threshold dependence. We examined whether continuous-IZ modeling provides complementary retrospective prioritization relative to calibrated binary classification in a highly imbalanced dataset. **Methods:** In this retrospective matched-data evaluation, we revisited a published ChEMBL-derived *P. aeruginosa* disk diffusion dataset using preserved training and locked external validation partitions. A calibrated support vector classifier using Molecular ACCess System keys (SVC/MACCS) provided conservative binary active calls. RegressionStack combined source-descriptor extreme gradient boosting (XGBoost) and ElasticNet regressors, Morgan-fingerprint random forest and gradient-boosting regressors, and a MACCS-key XGBoost regressor through an XGBoost meta-regressor to predict continuous IZ. **Results:** On the locked external set (*n* = 1130; 87 actives), SVC/MACCS achieved a positive predictive value (PPV) = 0.619, receiver operating characteristic area under the curve (ROC-AUC) = 0.857, precision–recall area under the curve (PR-AUC) = 0.479, and enrichment factor at 1% (EF@1%) = 7.58. RegressionStack achieved a mean absolute error (MAE) = 3.20 mm, ROC-AUC = 0.896, PR-AUC = 0.545, and EF@1% = 9.74. Neither paired permutation tests (ROC-AUC, *p* = 0.501; PR-AUC, *p* = 0.442) nor paired bootstrap confidence intervals resolved these differences. The y-randomization analyses supported non-random signals; scaffold-grouped validation retained early enrichment but showed reduced broader performance. The consensus-positive tier contained 27 actives among 35 nominations (PPV = 0.771). At measured IZ ≥ 30 mm, MAE increased to 9.46 mm, and all 30 compounds were underpredicted. **Conclusions:** Continuous-target modeling retained the IZ scale during training and generated a threshold-flexible predicted-IZ prioritization coordinate complementary to, but not statistically superior to, calibrated binary classification. The methodological contribution is a matched-data evaluation of complete workflows and their nomination behavior under the same partitions, yielding a retrospective compound-tiering scheme. Because the pipelines differed in architecture and molecular representation, their differences cannot be attributed solely to endpoint formulation. The workflows were not prospectively evaluated on compounds lacking pre-existing IZ measurements, and whether retrospective enrichment improves experimental hit discovery or reduces screening workload remains to be established.

## 1. Introduction

Antimicrobial resistance (AMR) continues to hinder the effectiveness of infectious disease treatment and remains a major global challenge for antibacterial discovery and public health. The most recent global burden analysis estimated that bacterial AMR was linked to an estimated 4.71 million deaths globally in 2021, including 1.14 million deaths caused directly by resistant bacterial infections [1].

Among Gram-negative pathogens, *Pseudomonas aeruginosa* (*P. aeruginosa*) is a clinically significant pathogen because of its ability to cause severe infections in vulnerable populations, including individuals with cystic fibrosis, burns, immunodeficiency, cancer, and chronic respiratory disease, and it remains a major cause of hospital-acquired infections [2,3]. The World Health Organization Bacterial Priority Pathogen List 2024 classifies carbapenem-resistant *P. aeruginosa* as a high-priority threat, emphasizing the urgent need for novel antibacterial agents and targeted public health interventions [4]. Eradicating this pathogen is exceptionally challenging because of its complex network of resistance mechanisms, which include low outer membrane permeability, active multidrug efflux, β-lactamase production, biofilm-associated tolerance, and rapid adaptive responses under antimicrobial pressure [3,5]. Together, these clinical and biological challenges render *P. aeruginosa* a compelling target for antibacterial discovery, highlighting the urgent need to identify and prioritize compounds.

Computational prioritization approaches offer a powerful means to narrow down antibacterial libraries before resource-intensive experimental screening. Among these approaches, ligand-based quantitative structure–activity relationship (QSAR) modeling provides a robust framework for linking molecular structures to biological activities [6,7]. By identifying key patterns across molecular descriptors, fingerprints, and experimentally derived activity data, QSAR models can effectively support the identification of promising candidate compounds from large chemical library collections. However, the practical utility of a QSAR model strongly depends on its intended decision-making context. In virtual screening, the primary objective is typically to nominate a selective list of high-probability candidates for experimental validation, rather than to assign equal predictive weight to every compound in a library [8,9]. Consequently, maximizing model interpretability and success requires careful optimization of endpoint definitions, data quality, chemical domain coverage, validation design, and screening-centric performance metrics [7,10]. In this screening paradigm, QSAR is intended not to replace microbiological testing but to enrich the follow-up pool with compounds more likely to exhibit the endpoint of interest; whether such enrichment reduces the experimental workload must ultimately be assessed prospectively.

Disk diffusion inhibition-zone (IZ) assays provide a widely adopted phenotypic measure of antibacterial activity. The standardized single-disk method introduced by Bauer, Kirby, Sherris, and Turck established the IZ diameter as an experimentally accessible readout of antimicrobial effects [11]. Modern disk diffusion testing remains highly valuable because of its reproducibility under standardized laboratory conditions [12]. However, the IZ diameter is not a direct measure of intrinsic molecular potency; rather, it represents a composite phenotype reflecting antibacterial activity alongside agar diffusion and physicochemical behaviors, including molecular weight, lipophilicity, charge state, and matrix binding [11,12,13]. In addition to these physicochemical dependencies, disk diffusion IZ measurements carry inherent technical noise. IZ diameters can be modulated by variations in inoculum density, agar depth and composition, disk application, incubation conditions, and the specific method—manual or automated—used to determine the zone edge [14]. While standardized protocols are specifically designed to minimize these sources of variation [15], this technical noise cannot be completely eliminated. Consequently, QSAR models trained on heterogeneous, publicly available IZ records should be interpreted as models of a noisy whole-cell phenotypic screening readout, rather than as direct predictors of intrinsic potency, minimum inhibitory concentrations (MICs), clinical susceptibility, or in vivo efficacy. While this multi-factorial nature makes the IZ a challenging endpoint for machine learning, it raises a critical, testable question: whether the original continuous diameter contains ranking-relevant information that is systematically lost when converted into a binary activity label.

Earlier QSAR studies targeting *P. aeruginosa* provided valuable insights but were largely constrained by their local or target-specific scope. Podunavac-Kuzmanović et al. utilized multiple linear regression and leave-one-out validation to model the MIC-derived activity of 14 substituted benzimidazoles, evaluating their approach on an external set of five structurally related analogues [16]. The authors restricted the applicability domain (AD) of their models to substituted 1-benzyl or 1-benzoylbenzimidazole derivatives, underscoring that these relationships were tailored for intra-class prediction rather than generalization across diverse chemotypes [16]. Similarly, Kadam and Roy investigated *P. aeruginosa* LpxC inhibitors across three distinct structural classes [17]. They observed that a global model pooled across all classes underperformed relative to cluster-specific models, a finding consistent with class-dependent SARs that are better captured locally. Furthermore, their endpoint was restricted to target-specific LpxC inhibition rather than a heterogeneous whole-cell antibacterial phenotype. Datar modeled inhibition-zone (IZ) diameters for a series of 15 indolylpyrimidines, again limiting inference to a small local chemical series [18]. More recently, Gajjar et al. expanded QSAR modeling of anti-*P. aeruginosa* activity but reported reduced external predictivity relative to internal validation, highlighting dataset expansion as a prerequisite for robust model generalization [19]. Taken together, these studies indicate that continuous antibacterial QSAR modeling is not inherently flawed; rather, earlier anti-*P. aeruginosa* models were primarily designed for localized chemotype or target-specific predictions, leaving broader external validation underexplored.

To address these limitations, Bugeac et al. subsequently established a substantially larger, ChEMBL-derived *P. aeruginosa* disk diffusion benchmark centered on binary activity classification [20]. Because the original IZ diameters and predefined training and external validation partitions were available, this benchmark also created an opportunity to examine the same phenotypic endpoint without first reducing it to a single activity label. The resulting methodological question is whether continuous-IZ prediction provides useful compound prioritization information beyond binary classification when both formulations are evaluated using the same compounds and data partitions. This question is relevant because antibacterial screening datasets are frequently imbalanced. Under severe class imbalance, conventional accuracy can be a misleading metric because a model can achieve a high score simply by assigning most compounds to the majority inactive class [21]. To address this problem, metrics derived from precision–recall (PR) analysis are widely considered more informative than receiver operating characteristic (ROC) curves, as precision directly isolates the fraction of the predicted positives that are true actives [21,22,23]. Recent virtual screening studies have emphasized positive predictive value (PPV) and top-k precision as practical indicators of hit-list utility [8,9], while enrichment-based metrics evaluate the critical early-recognition capability among top-ranked compounds [24]. These metrics are therefore appropriate for retrospective evaluation of nomination quality under class imbalance, although their prospective operational value requires experimental confirmation.

In the present study, we conducted a retrospective methodological evaluation of the *P. aeruginosa* disk diffusion QSAR benchmark established by Bugeac et al. [20]. The central question was whether modeling the original IZ measurements as a continuous endpoint preserves assay-scale prioritization information that is not fully represented by a single binary activity definition. By retaining the same compounds and predefined training and external validation partitions, the study provides a matched comparison of endpoint formulations rather than a comparison confounded by different datasets.

**Figure 1:**
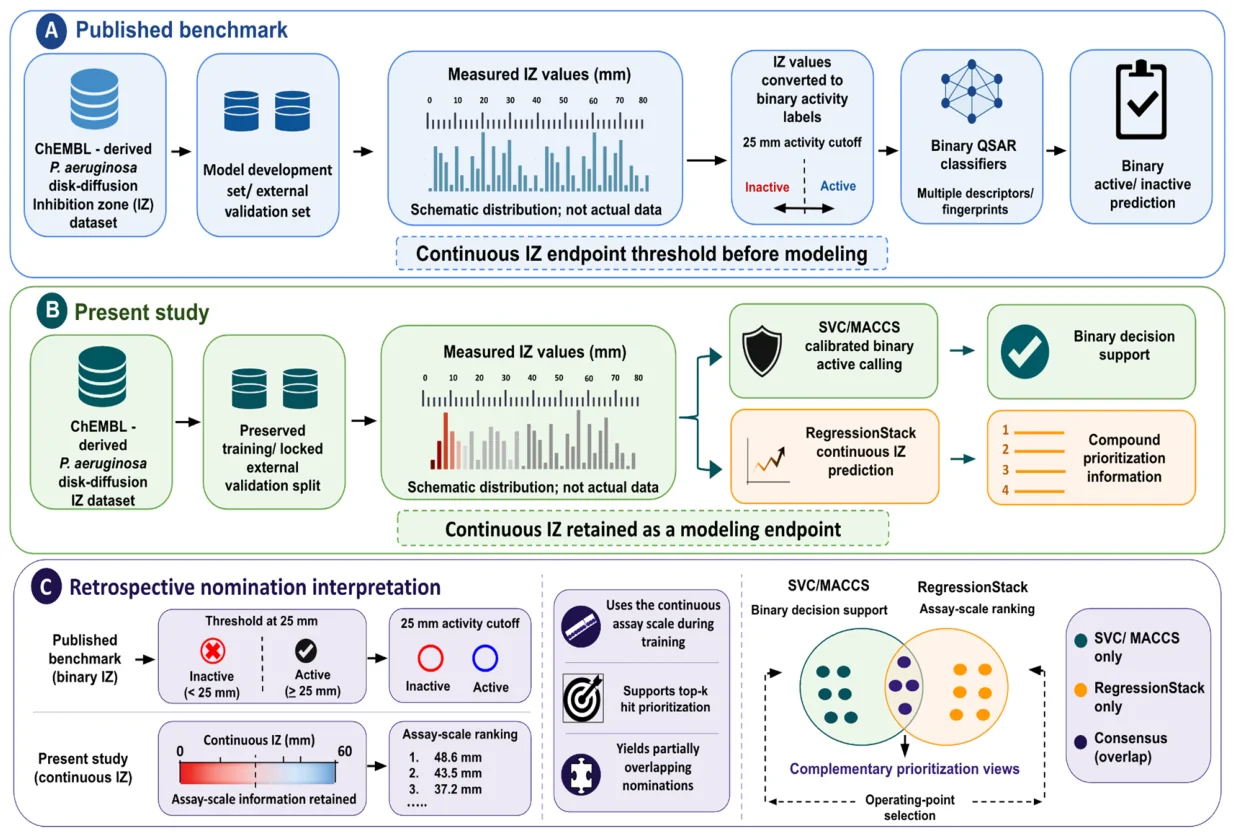
Matched-data evaluation of binary and continuous IZ QSAR workflows. The published *P. aeruginosa* disk diffusion benchmark converted experimentally measured IZ values into binary activity labels before classification modeling. In the present retrospective study, the binary benchmark role was retained through calibrated SVC/MACCS classification, whereas RegressionStack used the continuous-IZ diameter as its training target and generated assay-referenced predictions for ranking and threshold exploration. Both workflows were evaluated using the same compounds and preserved training and external validation partitions. Because the pipelines differ in endpoint formulation, model architecture, and molecular representation, the comparison evaluates their overall retrospective behavior and does not isolate endpoint formulation as the sole cause of observed differences. All distributions and numerical examples are schematic and do not represent study observations.

To evaluate this hypothesis, we established two distinct model roles. First, a calibrated support vector classifier (SVC) using Molecular ACCess System (MACCS) keys was used for conservative binary active calling under the benchmark threshold of IZ ≥ 25 mm. In parallel, a stacked regression framework (RegressionStack) was trained to predict continuous-IZ values as a threshold-flexible prioritization coordinate. Because conservative active calling and assay-scale prioritization are related but distinct virtual screening tasks, we investigated whether these two model outputs provided redundant or complementary decision information (Figure 1). The proposed framework positions continuous-IZ modeling neither as a replacement for calibrated binary QSAR classification nor as a direct predictor of in vivo efficacy, but as a complementary strategy for early-stage *P. aeruginosa* phenotypic-screening prioritization. The principal methodological contribution of this retrospective study is therefore a matched-data evaluation of two predefined modeling roles using the same experimentally measured disk diffusion endpoint, compound sets, and preserved training and external validation partitions, together with nomination-level analyses of their agreement and disagreement. The study evaluates retrospective predictive and prioritization behavior rather than prospective screening performance. No prospective experimental validation of model-nominated, previously unmeasured compounds was performed; all reported nomination metrics were calculated using compounds with existing experimentally measured IZ values.

## 2. Results

### 2.1. Dataset Composition and IZ Threshold Structure

**Figure 2:**
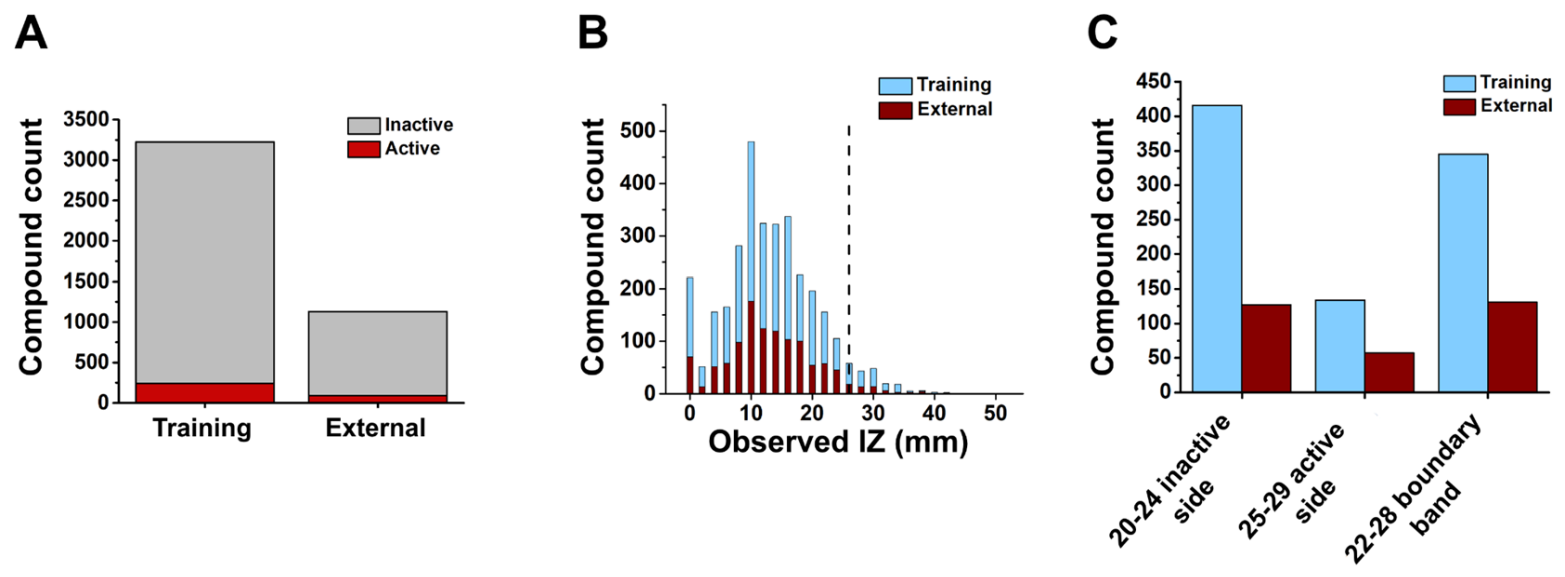
Dataset class imbalance and IZ endpoint distribution. (**A**) Active (IZ ≥ 25 mm) and inactive compound counts in the training and locked external validation sets; active prevalence was 7.44% and 7.70%, respectively. (**B**) Histogram of continuous-IZ values across both splits; the dashed vertical line marks the IZ = 25 mm binary activity threshold. (**C**) Counts of compounds in near-threshold IZ intervals, including the 20–24 mm inactive-side interval, the 25–29 mm active-side interval, and the 22–28 mm boundary band, illustrating the density of compounds close to the binary threshold.

Prior to model evaluation, the dataset composition and continuous-IZ distribution were characterized to define the class imbalance structure, support the selected evaluation metrics, and motivate the matched-data assessment of binary and continuous-target workflows. The training set contained 240 active compounds among 3226 molecules, whereas the locked external validation set contained 87 active compounds among 1130 molecules (Figure 2A), yielding active prevalence rates of 7.44% and 7.70%, respectively. This rare-positive structure makes ordinary accuracy misleading; a trivial all-inactive classifier would exceed 92% accuracy while failing to recover any active compounds. Therefore, performance evaluation emphasized PPV, precision–recall area under the curve (PR-AUC), early enrichment, and balanced accuracy. The PR-AUC was included because PR analysis is informative under class imbalance [21,22,23], whereas PPV and top-k precision reflect virtual screening hit-list utility [8,9], and enrichment assesses early recognition among top-ranked compounds [24].

Analysis of the IZ distributions further confirmed that the endpoint remained continuous despite the binary activity definition used for benchmarking (Figure 2B). Although the IZ ≥ 25 mm cutoff was retained for comparability with the source benchmark [20], this threshold created a near-boundary region where compounds with similar measured IZ values could receive opposite binary labels. Within the training set, 416 compounds were distributed in the 20–24 mm interval, and 134 were distributed in the 25–29 mm interval. In the locked external validation set, the corresponding counts were 127 and 57 (Figure 2C). This boundary density motivated evaluating the retrospective prioritization behavior of a workflow trained on continuous-IZ values alongside a workflow trained on the corresponding binary labels.

### 2.2. External Validation of the Calibrated SVC/MACCS Binary Classifier

**Figure 3:**
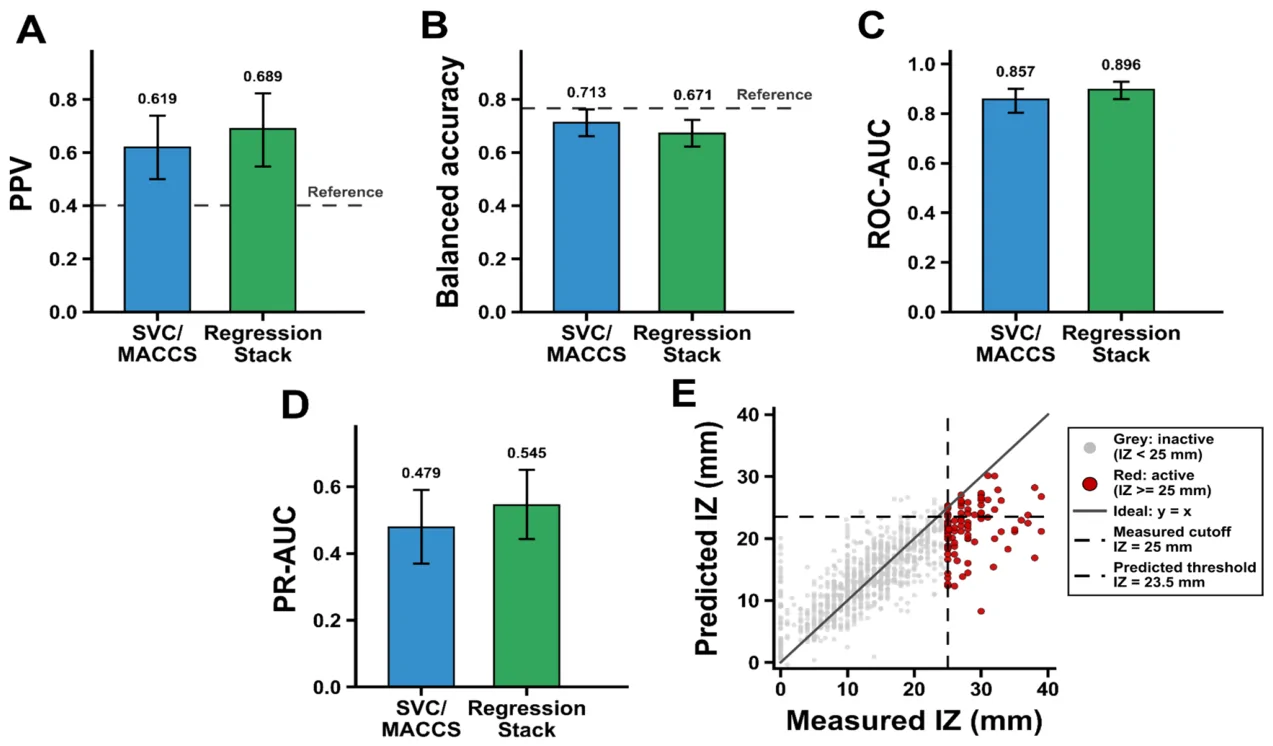
External validation performance with bootstrap uncertainty (locked holdout; *n* = 1130; 87 actives). Point estimates and 95% bootstrap confidence intervals from 1000 resamples are shown for (**A**) PPV, (**B**) balanced accuracy, (**C**) ROC-AUC, and (**D**) PR-AUC for SVC/MACCS and RegressionStack. Dashed horizontal lines in panels A and B indicate aggregate PPV and balanced accuracy values reported by Bugeac et al. [20]. (**E**) Measured versus predicted IZ values for RegressionStack; the vertical dashed line marks the measured IZ = 25 mm activity threshold, and the horizontal dashed line marks the predicted IZ = 23.50 mm operating threshold.

The SVC/MACCS classifier was evaluated on the locked external set to establish a conservative calibrated binary comparator under the published IZ greater than or equal to the 25 mm activity definition. At the out-of-fold-selected probability threshold of ≥0.360, the model selected 63 external compounds as active, yielding 39 true positives (TPs) and 24 false positives (FPs) (PPV = 0.619; Figure 3A). Within the locked retrospective benchmark, the 63 nominated compounds contained 39 compounds with measured IZ ≥ 25 mm, corresponding to PPV = 0.619 and approximately eightfold enrichment relative to the 7.70% active prevalence. For context, the previously published benchmark reported an aggregate PPV of 0.400 [20]; however, because the original per-compound predictions and operating thresholds were unavailable, this comparison remains contextual rather than threshold matched.

The model showed a sensitivity of 0.448, specificity of 0.977, and balanced accuracy of 0.713 (Figure 3B). This high specificity is consistent with the conservative nature of the selected threshold, which was optimized to prioritize precision over recall when the experimental follow-up capacity was constrained. The threshold-independent discrimination metrics were consistent with the threshold-dependent classification results. The SVC/MACCS achieved ROC-AUC = 0.857 and PR-AUC = 0.479 on the locked external validation set (Figure 3C,D). These threshold-independent metrics extend the source benchmark, which reports threshold-dependent classification metrics [20]. Early-recognition metrics were also consistent with useful active enrichment near the top of the prioritized list, with enrichment factor in the top 1% (EF@1%) = 7.58 and secondary Boltzmann-enhanced discrimination of receiver operating characteristic (BEDROC) = 0.615 (Figure S1A,B). Probability calibration on the locked external set was favorable, with the expected calibration error (ECE) = 0.0116 (Figure S2A,B). Together, these results define SVC/MACCS as a conservatively calibrated active calling model that provides a fixed binary reference point for evaluating complementary continuous IZ prioritization.

Descriptor-representation sensitivity analysis showed mixed metric-level changes rather than a systematic advantage for Morgan fingerprints. SVC/Morgan achieved ROC-AUC = 0.865 and EF@1% = 9.74, compared with 0.857 and 7.58, respectively, for SVC/MACCS. However, SVC/Morgan showed a lower operating threshold PPV (0.554 versus 0.619), balanced accuracy (0.666 versus 0.713), and PR-AUC (0.445 versus 0.479). PPV-25 was slightly higher for SVC/Morgan (0.680 versus 0.640), whereas PPV-50 and PPV-100 were lower (0.600 versus 0.620 and 0.420 versus 0.490, respectively). All computed paired bootstrap confidence intervals for the between-representation differences included zero, indicating that the observed differences were not statistically resolved (Figure S3; Table S1). Thus, the primary SVC/MACCS comparator was not demonstrably disadvantaged by its molecular representation.

### 2.3. RegressionStack Continuous Prediction, Error Structure, and Prioritization Performance

#### 2.3.1. Continuous Prediction Accuracy and Residual Structure

To characterize the retrospective behavior of the continuous-target workflow, RegressionStack was evaluated for continuous-IZ prediction, ranking, and threshold-based nomination. Its predicted IZ was interpreted as an assay-referenced prioritization coordinate rather than an exact replacement for experimental IZ measurement or evidence of superiority over SVC/MACCS.

**Figure 4:**
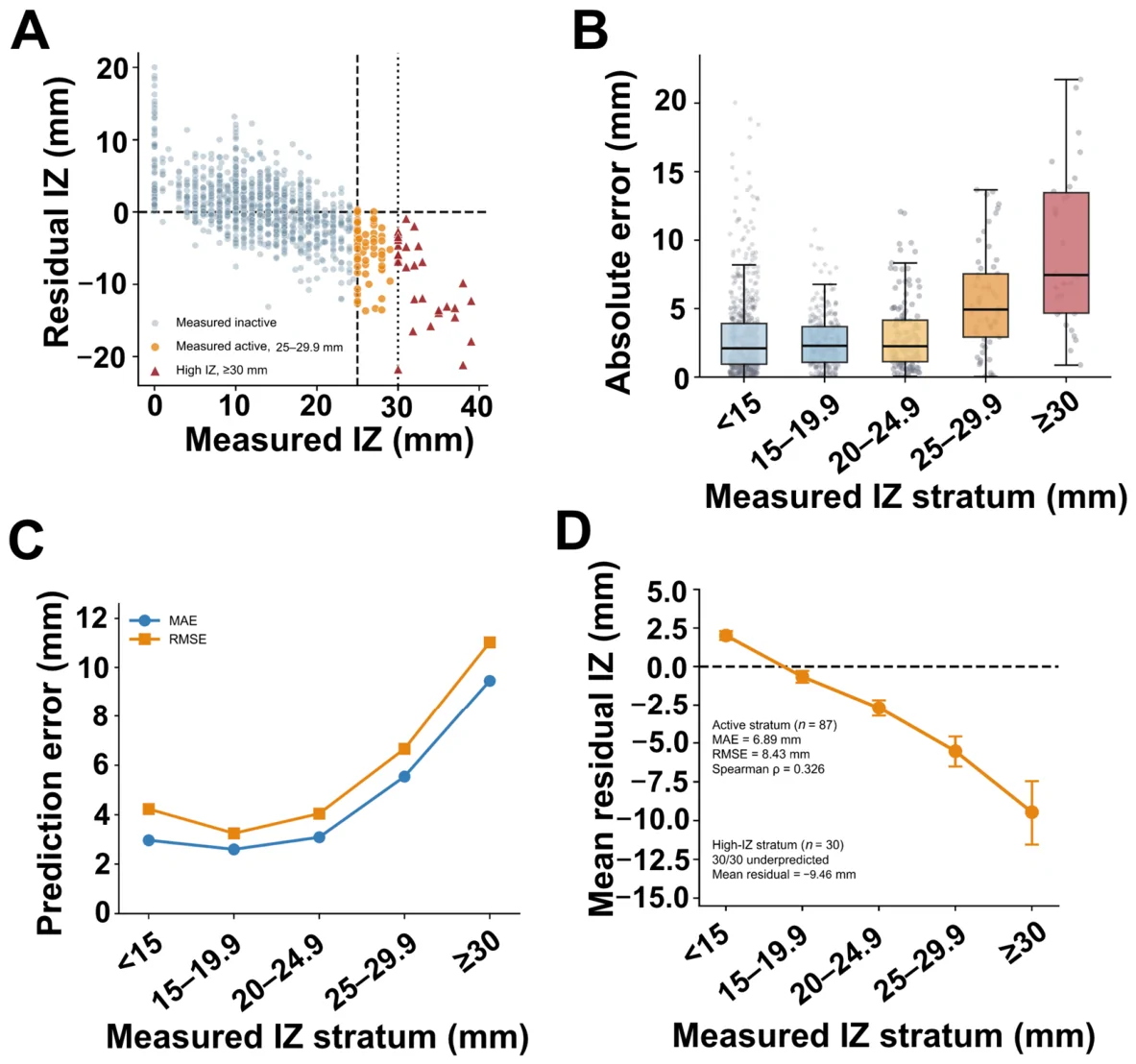
RegressionStack residual and prediction error structure across the measured IZ range on the locked external validation set (*n* = 1130). (**A**) Signed residuals, defined as predicted minus measured IZ diameter, versus measured IZ. Horizontal and vertical reference lines indicate zero residual and measured IZ thresholds of 25 and 30 mm, respectively. (**B**) Absolute error distributions across measured IZ strata; boxes show medians and interquartile ranges, with individual observations overlaid. (**C**) Mean absolute error (MAE) and root mean square error (RMSE) by IZ stratum. (**D**) Mean signed residual by IZ stratum; negative values indicate underprediction. Error bars represent percentile-based 95% bootstrap confidence intervals from 10,000 within-stratum resamples. Among measured actives (IZ ≥ 25 mm; *n* = 87), MAE was 6.89 mm, RMSE was 8.43 mm, and Spearman’s ρ was 0.326. All 30 compounds with measured IZ ≥ 30 mm were underpredicted, with a mean residual of −9.46 mm.

The RegressionStack predicted external IZ values with root mean square error (RMSE) = 4.48 mm, mean absolute error (MAE) = 3.20 mm, R^2^ = 0.618, Pearson r = 0.787, Spearman ρ = 0.792, and concordance correlation coefficient (CCC) = 0.758 (Figure 3E; Table S2). The training-mean IZ baseline gave RMSE = 7.25 mm and MAE = 5.70 mm, indicating that RegressionStack captured structure–IZ associations beyond a trivial central-tendency predictor. However, residual diagnostics showed that the prediction error was not uniform across the measured IZ range (Figure 4). The error increased in the higher-IZ strata: MAE was 3.09 mm for measured IZ values of 20–24.9 mm, 5.54 mm for 25–29.9 mm, and 9.46 mm for measured IZ values greater than or equal to 30 mm (Table S3). In the high-IZ stratum, all 30 compounds were underpredicted, with an RMSE of 11.03 mm and mean residual of −9.46 mm. These findings indicate a systematic high-IZ underprediction and support the interpretation of the predicted IZ as a coarse prioritization coordinate rather than a precise millimeter-level estimate.

The active stratum, defined as measured IZ greater than or equal to 25 mm, was also evaluated separately to assess within-active ordering. In this subset, RegressionStack showed MAE = 6.89 mm, RMSE = 8.43 mm, mean residual = −6.88 mm, median absolute error = 5.85 mm, Pearson’s r = 0.265, and Spearman’s ρ = 0.326 (Table S3). Thus, the predicted IZ retained only modest within-active ordering information. This result further supports the use of RegressionStack for coarse threshold exploration and screening triage rather than fine-grained ranking among active compounds.

#### 2.3.2. Calibration Sensitivity and Specialist-Regressor Comparison

Because the residual analysis showed a systematic high-IZ underprediction, we next tested whether this pattern could be corrected by training-only monotonic calibration. Four candidate mappings were evaluated using training out-of-fold prediction pairs only: identity, ordinary least squares linear calibration, robust Huber calibration, and isotonic calibration. Identity calibration achieved the lowest internal macro-bin MAE, with macro-bin MAE = 5.445 mm compared with 5.486 mm for Huber, 5.508 mm for ordinary least squares, and 5.534 mm for isotonic calibration (Figure S4). Therefore, no post hoc recalibration was applied to locked external predictions. The high-IZ underprediction pattern remained unchanged, indicating that the error was not a simple monotonic scale-calibration artifact.

Among the first-level specialist regressors, the extreme gradient boosting (XGBoost) regressor trained on MACCS keys was the best single model by MAE, with MAE = 3.38 mm and R^2^ = 0.585 (Table S4). Stacking reduced the MAE by 5.3% relative to the XGBoost/MACCS specialist model. This improvement was modest and primarily reflected continuous-target error reduction; the stacked model did not dominate every downstream discrimination or enrichment metric, as XGBoost/MACCS showed marginally higher ROC-AUC and PR-AUC in the specialist comparison. Because RegressionStack was designed as a continuous prioritization model, continuous-target error was treated as the primary criterion for stacking utility, while binary discrimination and enrichment metrics were evaluated after comparing the predicted IZ with the measured IZ greater than or equal to a 25 mm activity label.

#### 2.3.3. Ranking, Early Enrichment, and Operating Threshold Behavior

**Figure 5:**
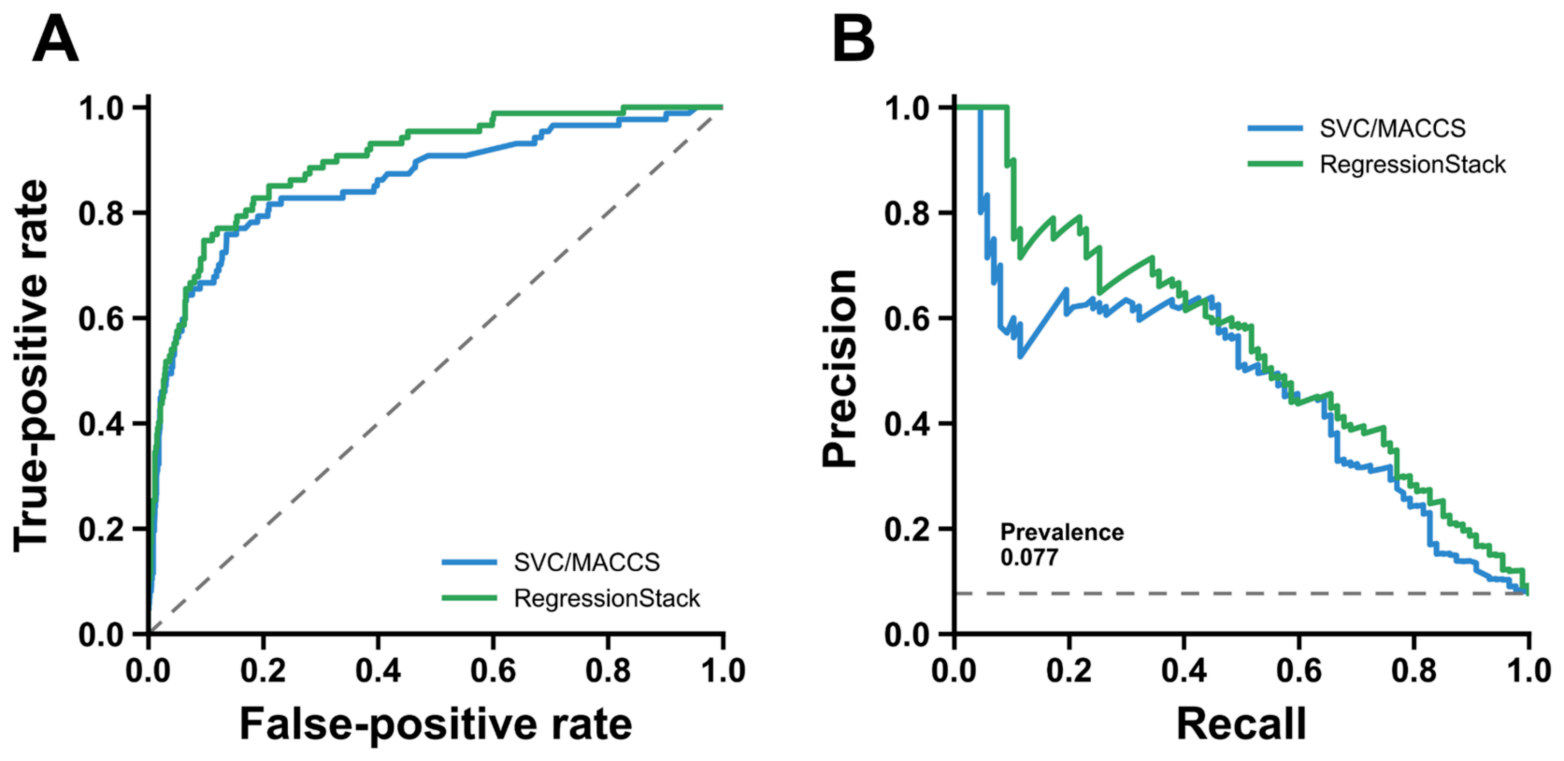
Threshold-independent ranking performance on the locked external holdout (*n* = 1130; 87 actives). (**A**) ROC curves and (**B**) PR curves for SVC/MACCS and RegressionStack. Diagonal and horizontal dashed lines indicate random classifier baselines (ROC-AUC = 0.500; PR-AUC = active prevalence = 0.077). RegressionStack point estimates exceeded those of SVC/MACCS, but paired permutation tests did not support statistically significant differences (ROC-AUC: *p* = 0.501; PR-AUC: *p* = 0.442).

Using the predicted IZ for threshold-independent prioritization, RegressionStack achieved ROC-AUC = 0.896 and PR-AUC = 0.545 on the locked external set, exceeding the corresponding SVC/MACCS point estimates shown in the external-performance summary (Figure 3C,D). These differences are interpreted descriptively here, while formal paired model-difference uncertainty is reported in Section 2.4. The full ROC and PR curves are shown in Figure 5A and Figure 5B, respectively. Early-recognition analysis yielded EF@1% = 9.74 and secondary BEDROC = 0.657 (Figure S1A,B). In the fixed-size nomination analysis, RegressionStack recovered 19, 33, and 49 actives among the top 25, 50, and 100 prioritized compounds, respectively, corresponding to PPV-25 = 0.760, PPV-50 = 0.660, and PPV-100 = 0.490 (Table S5).

**Figure 6:**
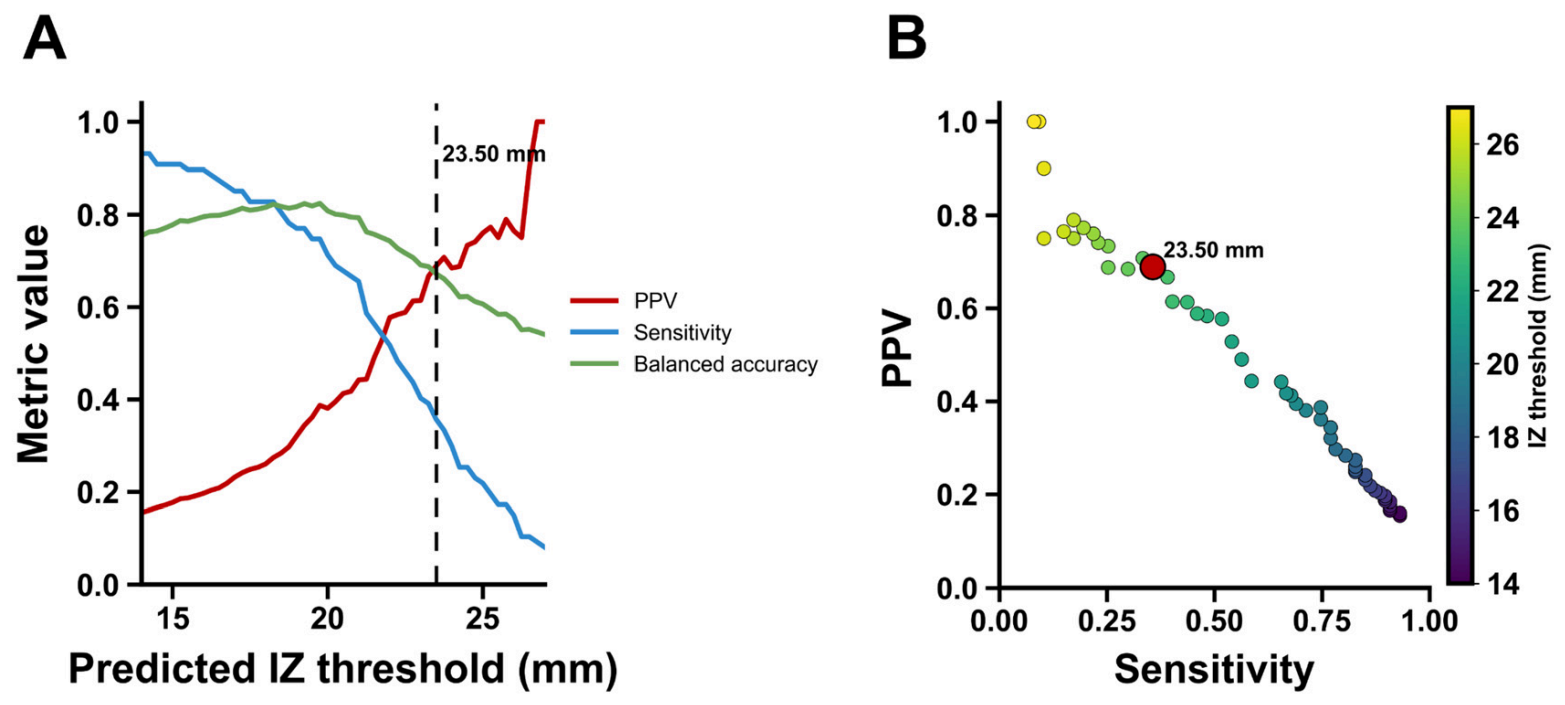
Threshold-dependent operating behavior of RegressionStack. (**A**) PPV, sensitivity, and balanced accuracy as functions of predicted-IZ threshold on the locked external set; the vertical dashed line marks the out-of-fold-selected operating threshold (23.50 mm). (**B**) PR frontier as the predicted-IZ threshold varies, with points colored by predicted-IZ threshold and the 23.50 mm operating point highlighted.

Because RegressionStack generated outputs on the IZ scale, its predicted-value threshold could be varied after training to characterize retrospective PPV–sensitivity trade-offs at different nomination-set sizes. RegressionStack supported a continuous spectrum of operating points across the predicted-IZ scale, with PPV, sensitivity, and balanced accuracy varying as the predicted-IZ threshold changed on the locked external set (Figure 6A). The corresponding PR frontier shows the trade-off between hit-list precision and active recovery as the threshold varies, with the 23.50 mm operating point highlighted (Figure 6B). The out-of-fold-selected threshold of predicted IZ ≥ 23.50 mm selected 45 compounds with 31 TP and 14 FP (PPV = 0.689, sensitivity = 0.356, specificity = 0.987, and balanced accuracy = 0.671). At the predicted-IZ threshold corresponding to the measured activity boundary of 25 mm, the selected set contracted to 25 compounds with 19 TP and PPV = 0.760, at the cost of missing 68 of 87 external actives. Expressing these operating points in millimeters provides an assay-referenced interpretation of the retrospective threshold trade-off, although predicted IZ should not be interpreted as a precise experimental measurement.

### 2.4. Model-Difference Uncertainty and Y-Randomization Robustness

Because point estimates from a single locked external set containing only 87 actives are subject to sampling variability, bootstrap resampling was used to quantify performeruginosaance uncertainty. Bootstrap resampling showed that both models retained enrichment above the 7.70% external active prevalence across resampled estimates (Figure 3A–D) [25]. For SVC/MACCS, the 95% bootstrap confidence intervals were 0.500–0.738 for PPV, 0.661–0.763 for balanced accuracy, 0.803–0.901 for ROC-AUC, and 0.370–0.590 for PR-AUC. For RegressionStack, the corresponding intervals were 0.548–0.822, 0.622–0.723, 0.859–0.928, and 0.443–0.650 for PPV, balanced accuracy, ROC-AUC, and PR-AUC, respectively.

**Figure 7:**
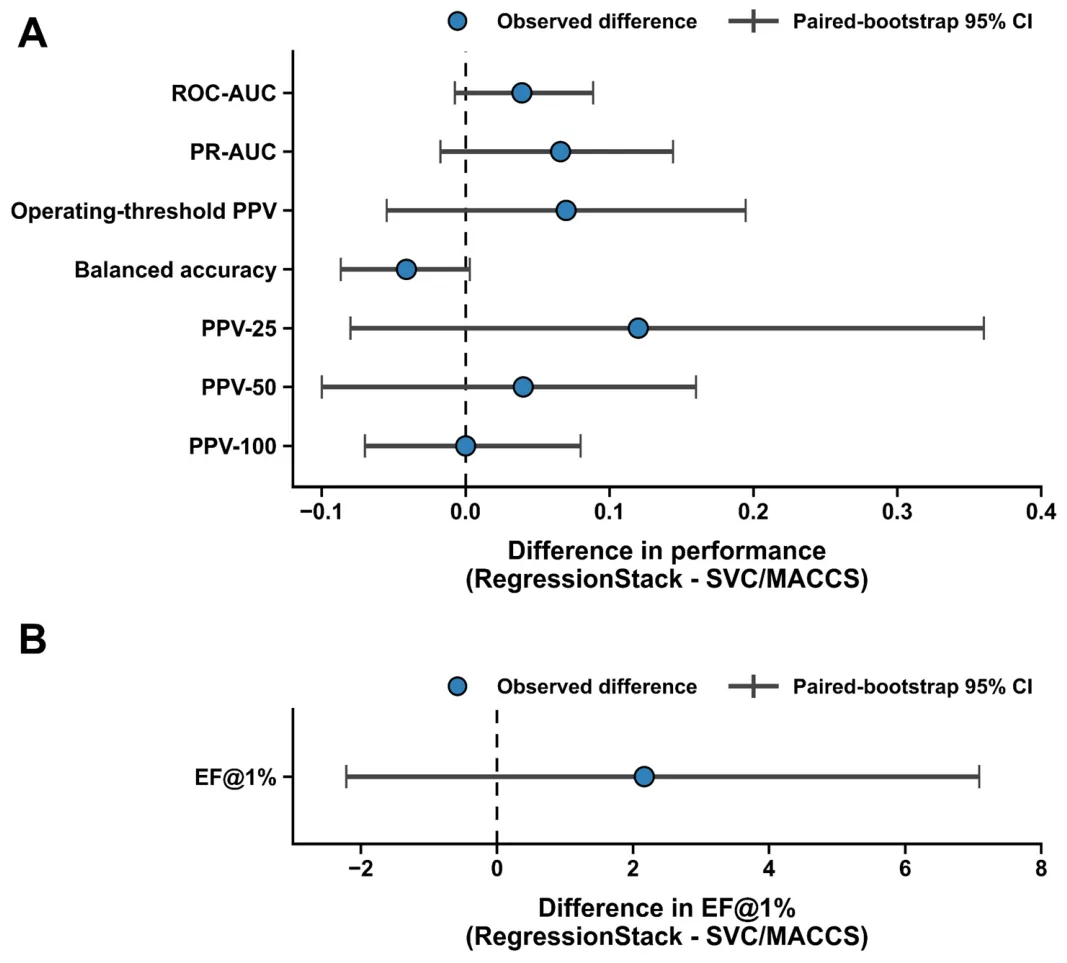
Paired bootstrap uncertainty in external validation performance differences between RegressionStack and SVC/MACCS. Differences were defined as RegressionStack minus SVC/MACCS. (**A**) Observed differences in ROC-AUC, PR-AUC, operating threshold PPV, balanced accuracy, and PPV at fixed nomination sizes of 25, 50, and 100 compounds. (**B**) Observed difference in EF@1%, displayed separately because this metric is expressed in fold-enrichment units. Symbols denote point estimates, horizontal bars denote percentile-based 95% confidence intervals obtained from 10,000 paired bootstrap resamples using identical compound indices for both models, and the dashed vertical line denotes the null value of zero. Confidence intervals spanning zero indicate that the corresponding between-model difference was not statistically resolved.

RegressionStack had descriptively higher point estimates than SVC/MACCS for ROC-AUC, PR-AUC, EF@1%, threshold PPV, PPV-25, and PPV-50, whereas SVC/MACCS had higher sensitivity and balanced accuracy at the selected operating threshold. Paired permutation tests did not establish statistically significant threshold-independent discrimination differences between the models (ROC-AUC: *p* = 0.501; PR-AUC: *p* = 0.442). Paired bootstrap confidence intervals for RegressionStack minus SVC/MACCS also included zero for all evaluated model-difference metrics (Figure 7). The observed differences were ΔROC-AUC = 0.039, 95% CI −0.007 to 0.089; ΔPR-AUC = 0.066, 95% CI −0.018 to 0.144; Δthreshold PPV = 0.070, 95% CI −0.055 to 0.195; Δbalanced accuracy = −0.041, 95% CI −0.087 to 0.003; ΔPPV-25 = 0.120, 95% CI −0.080 to 0.360; ΔPPV-50 = 0.040, 95% CI −0.100 to 0.160; ΔPPV-100 = 0.000, 95% CI −0.070 to 0.080; and ΔEF@1% = 2.165, 95% CI −2.216 to 7.089. These results support the descriptive differences between the models but do not establish the model-wide superiority of RegressionStack.

**Figure 8:**
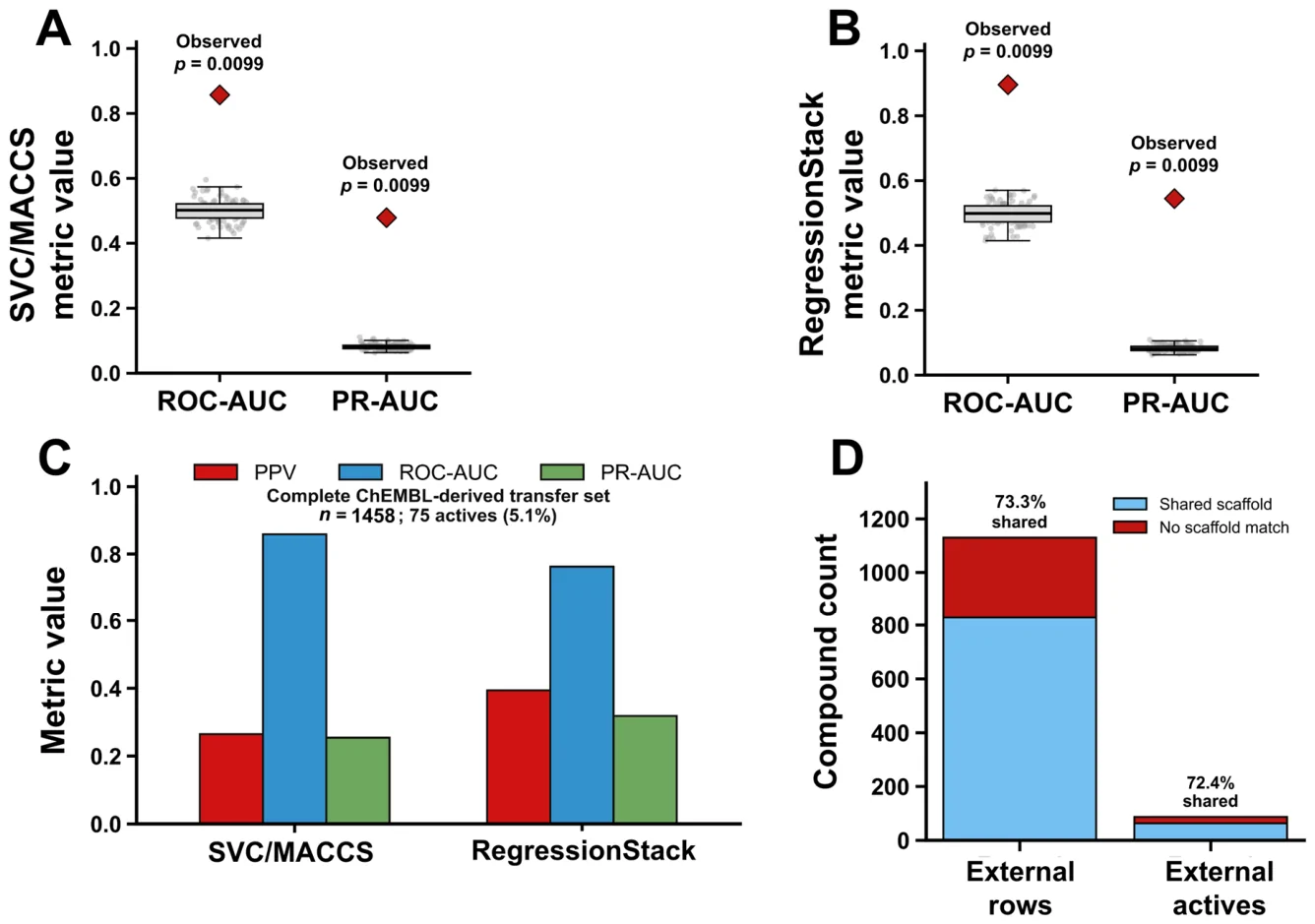
Robustness and cross-dataset transfer analyses. The y-randomization null distributions (100 permutations) for (**A**) SVC/MACCS and (**B**) RegressionStack; red diamonds mark observed external ROC-AUC and PR-AUC values. No permuted model matched or exceeded either metric (empirical *p* = 0.0099). (**C**) Transfer performance of both models on the ChEMBL-derived disk diffusion set (*n* = 1458; 75 actives; 5.1% prevalence; zero canonical SMILES overlap with source-paper compounds); metrics are shown as PPV, ROC-AUC, and PR-AUC. (**D**) Bemis–Murcko scaffold overlap between training and external compounds; 828 of 1130 external compounds (73.3%) shared a training scaffold, characterizing the holdout as a defined-domain benchmark.

The y-randomization was then used to test whether the observed performance exceeded the randomized label or randomized target null distributions [26,27]. For SVC/MACCS, the observed ROC-AUC of 0.857 exceeded the permuted-label null mean of 0.501 and the 95th percentile of 0.562. The observed PR-AUC of 0.479 exceeded the null mean of 0.081 and 95th percentile of 0.098 (Figure 8A). RegressionStack showed the same pattern under continuous-target randomization: the observed ROC-AUC of 0.896 and PR-AUC of 0.545 exceeded the null means of 0.497 and 0.082 and the 95th percentiles of 0.553 and 0.100, respectively (Figure 8B). Across all 100 retrained y-randomization runs, no permuted model matched or exceeded the observed ROC-AUC, PR-AUC, EF@1%, or BEDROC for either model (Figure 8A,B and Figure S1A,B). With a plus-one empirical correction, *p* = 0.0099 represents the resolution limit of the experiment. These results support the non-random structure–activity signal in both models, while model-difference analyses support a complementary rather than hierarchical interpretation.

### 2.5. ChEMBL-Derived Transfer and Descriptor-Reconstruction Sensitivity

To assess cross-dataset transfer beyond the source-paper holdout, an independently assembled ChEMBL-derived endpoint-matched dataset with zero exact canonical SMILES overlap was evaluated in a more chemically and assay-condition heterogeneous context. This analysis was treated as a transfer and heterogeneity stress test rather than validation under a single controlled experimental protocol.

The ChEMBL-derived disk diffusion/IZ set contained 1458 compounds and 75 actives, corresponding to 5.1% active prevalence [28]. An exact overlap analysis based on canonical SMILES confirmed that no compounds were shared with the source-paper compound dataset. Without model refitting, SVC/MACCS achieved ROC-AUC = 0.858, PR-AUC = 0.254, and EF@1% = 5.18 (Figure 8C). The ROC-AUC of 0.858 was nearly identical to the source-holdout value of 0.857, indicating the preservation of threshold-independent discrimination under endpoint-matched transfer conditions. The PR-AUC of 0.254 appears lower than the source-holdout value of 0.479; however, this comparison must account for the difference in active prevalence. Because the random classifier PR-AUC baseline equals active prevalence, the relevant comparison is fold enrichment over baseline: SVC/MACCS achieved approximately 6.22-fold enrichment on the source holdout (0.479/0.077) and approximately 4.98-fold enrichment on the ChEMBL-derived set (0.254/0.051). The partial reduction in the prevalence-normalized PR-AUC was consistent with the greater chemical and assay condition heterogeneity in the ChEMBL-derived set. At the locked binary threshold, SVC/MACCS produced 18 TP, 50 FP, and 57 false negatives, corresponding to PPV = 0.265 and balanced accuracy = 0.602. Thus, the source-holdout operating threshold did not transfer directly, even though the threshold-independent discrimination was retained.

The RegressionStack was evaluated using the Mordred-reconstructed source descriptors [29]. Agreement analysis on the source-paper external validation set showed that the mean absolute difference between the reconstructed and saved RegressionStack predictions was 0.063 mm, with a maximum absolute difference of 1.736 mm; ROC-AUC changed by less than 0.001, and PR-AUC changed by approximately 0.010 (Table S6). On the complete ChEMBL-derived set, RegressionStack achieved ROC-AUC = 0.763, PR-AUC = 0.318, EF@1% = 12.96, PPV = 0.394, sensitivity = 0.347, specificity = 0.971, and balanced accuracy = 0.659 at a predicted IZ greater than or equal to 23.50 mm. Top-k analysis further recovered 12, 19, and 32 actives among the top 25, 50, and 100 prioritized compounds, respectively, against random expectations of 1.3, 2.6, and 5.1, corresponding to enrichment factors (EFs) of 9.33, 7.39, and 6.22 (Table S5). Collectively, these findings support retention of threshold-independent prioritization and early enrichment performance under descriptor reconstruction, while the descriptor-drift qualification limits overinterpretation of absolute ChEMBL-transfer metric values.

### 2.6. Scaffold-Based Generalization and Applicability Domain Analysis

#### 2.6.1. Repeated Scaffold-Grouped Validation

To evaluate whether model performance persisted when related molecular scaffolds were prevented from appearing in both training and held-out folds, SVC/MACCS and RegressionStack were assessed using repeated scaffold-grouped validation. Both models retained early enrichment above random expectation under scaffold separation, with EF@1% = 6.52 ± 0.71 for SVC/MACCS and 8.28 ± 1.18 for RegressionStack (Figure S5; Table S7).

Broader performance estimates were lower under scaffold-grouped validation than under the original standard out-of-fold procedure. For SVC/MACCS, mean ROC-AUC was 0.741 ± 0.009, PR-AUC was 0.288 ± 0.006, PPV was 0.392 ± 0.016, and balanced accuracy was 0.603 ± 0.025, compared with standard out-of-fold values of 0.825, 0.393, 0.519, and 0.662, respectively. For the near-exact fixed-architecture RegressionStack analysis, mean MAE was 4.36 ± 0.06 mm, R^2^ was 0.388 ± 0.018, ROC-AUC was 0.784 ± 0.013, and PR-AUC was 0.318 ± 0.016, compared with standard out-of-fold values of 3.51 mm, 0.560, 0.860, and 0.408, respectively. The active-stratum MAE increased to 11.16 ± 0.11 mm, and the high-IZ MAE increased to 14.12 ± 0.11 mm. Thus, active enrichment persisted under scaffold separation, whereas broader discrimination and continuous IZ accuracy were scaffold-sensitive.

#### 2.6.2. Scaffold Overlap, Scaffold-Novel Performance, and AD

Direct scaffold overlap analysis of the locked external validation set provided additional context for these findings. Among the 1130 external compounds, 828 (73.3%) shared a Bemis–Murcko framework with at least one training compound; among the 87 external actives, 63 (72.4%) shared a training scaffold (Figure 8D). Therefore, the source-paper external validation set should be interpreted primarily as a defined-domain benchmark rather than a broad scaffold-novel extrapolation test.

In the scaffold-novel subset of the existing locked external split, comprising 302 compounds and 24 actives, SVC/MACCS nominated 11 compounds, including nine TP and two FP, corresponding to PPV = 0.818, ROC-AUC = 0.775, and PR-AUC = 0.482. RegressionStack nominated nine compounds, including eight TP and one FP, with PPV = 0.889, ROC-AUC = 0.865, and PR-AUC = 0.554 (Table S8). Because these estimates were based on small numbers of scaffold-novel actives and predicted positives, they were interpreted descriptively and should not be considered equivalent to the repeated scaffold-grouped validation results.

**Figure 9:**
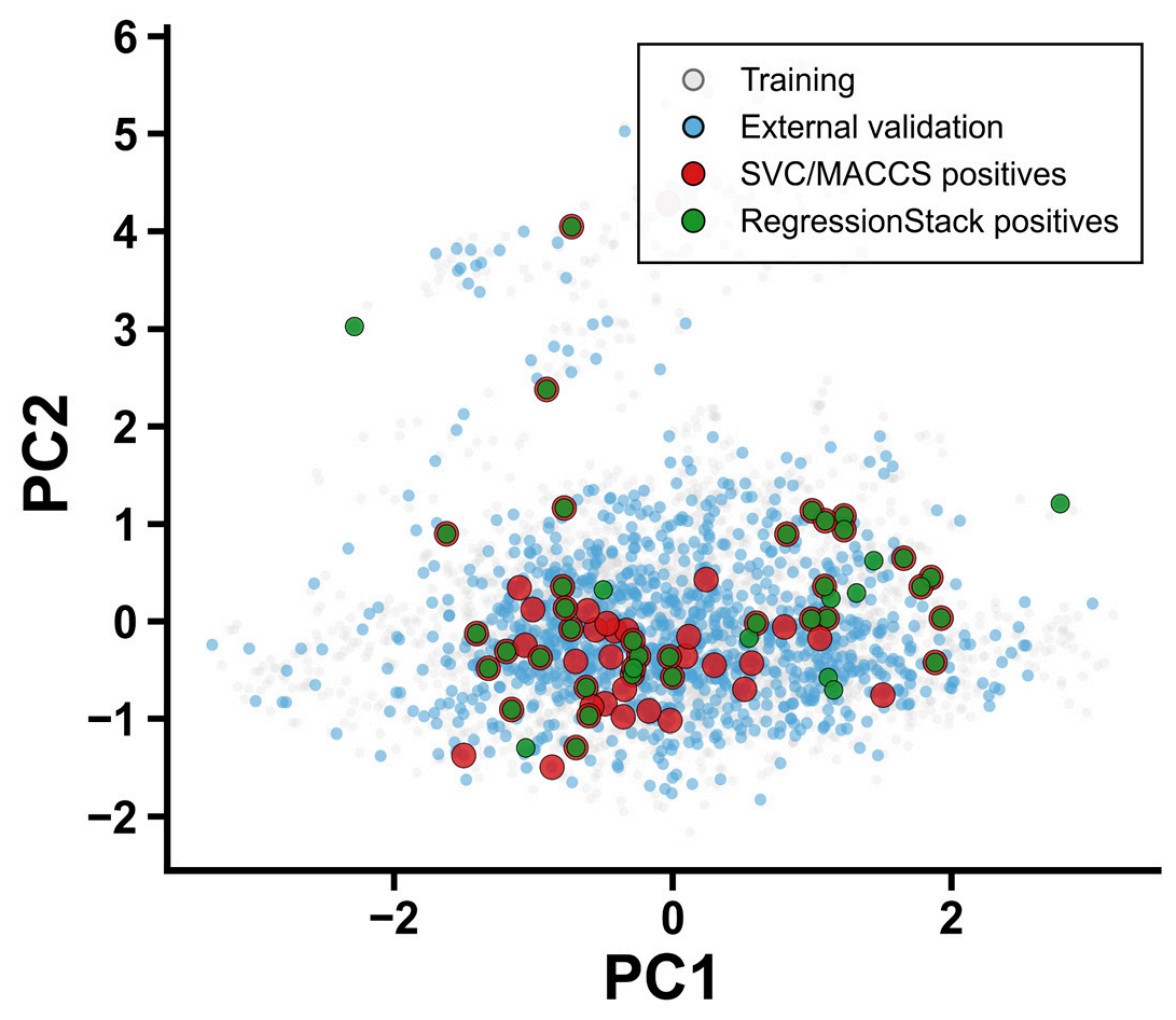
Chemical space structure and applicability domain context for external validation. PCA projection of Morgan fingerprints showing training compounds and all locked external validation compounds. SVC/MACCS-predicted positives at the probability ≥ 0.360 operating threshold (red dots; *n* = 63) and RegressionStack-predicted positives at the predicted IZ ≥ 23.50 mm operating threshold (green dots; *n* = 45) are overlaid on the training/external chemical space projection. The distribution shows that nominations from both models were embedded within the represented chemical space; all predicted positives fell within the Morgan/Tanimoto-defined applicability domain threshold of 0.474.

Chemical space PCA further supported the defined domain interpretation (Figure 9). Training and external compounds occupied overlapping fingerprint spaces, and the predicted positives from both models were embedded within this chemical domain. Using the Morgan/Tanimoto AD threshold of 0.474, 1095 of the 1130 external compounds were classified as inside-domain and 35 as outside-domain. All predicted positives from both SVC/MACCS and RegressionStack fell inside the defined AD; consequently, the outside-domain PPV was not estimable and is reported descriptively in Table S9.

### 2.7. Structural Feature Attribution for the SVC/MACCS Classifier

To provide structural transparency for the SVC/MACCS classifier, SHAP analysis was applied to the MACCS key representation [30]. High-attribution keys (Table S10) were mapped to chemically plausible feature classes, including heteroatom-containing fragments, ring-associated motifs, polar or ionizable groups, and halogenated substructures. The representative high-attribution keys are listed in Table S10, along with their MACCS definitions and structural interpretations. These features may correlate with the physicochemical properties relevant to antibacterial activity and disk diffusion behavior, including polarity, ionization state, molecular size, compound accumulation, and diffusion constraints [11,13,31,32,33,34,35].

This feature attribution was interpreted as associative rather than mechanistic because SHAP values describe model-specific contributions to predictions, not causal antibacterial mechanisms. Because disk diffusion IZ values reflect both the antibacterial effect and physicochemical behavior in agar, the identified features may encode a mixture of activity- and diffusion-relevant properties [11,13,18]. Therefore, SHAP analysis provides model transparency and hypotheses for follow-up but not empirical proof of the mechanism of action.

### 2.8. Tiered Nomination Overlap, Label-Permutation Analysis, and Threshold Margins

**Figure 10:**
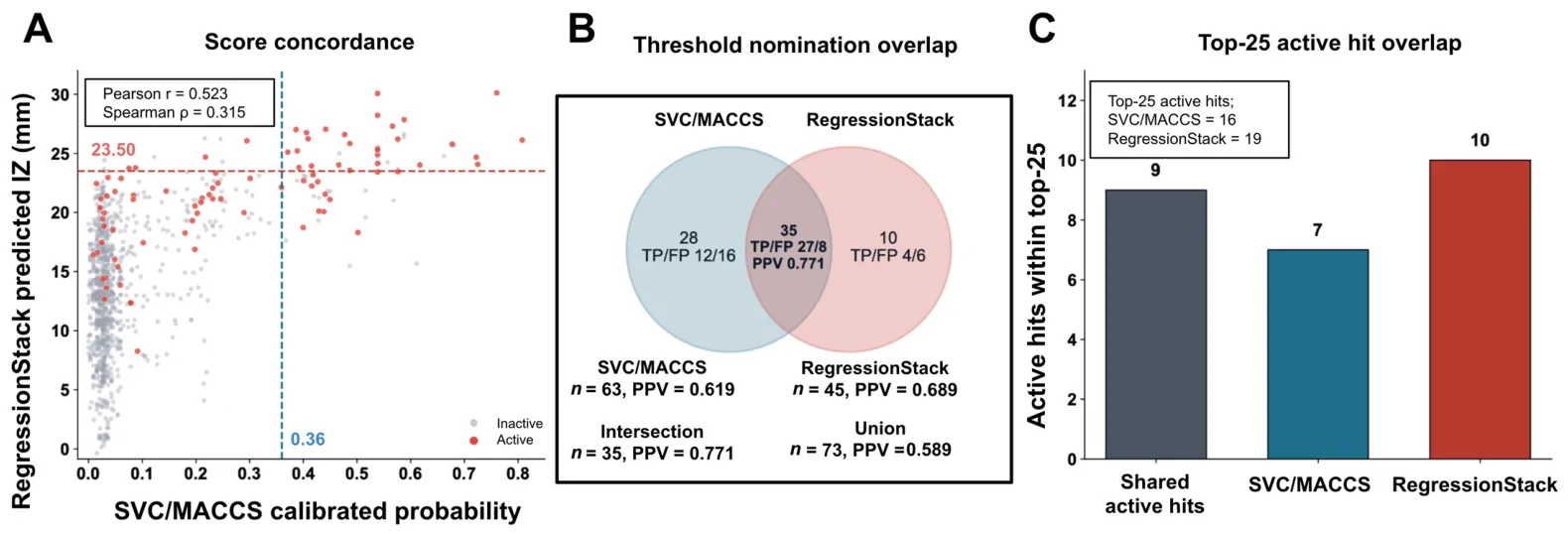
Tiered nomination overlap and operational complementarity on the locked external validation set. (**A**) Score concordance between SVC/MACCS calibrated probabilities and RegressionStack predicted-IZ values. Point color indicates measured activity status, and dashed lines mark the predefined operating thresholds of SVC/MACCS probability greater than or equal to 0.360 and RegressionStack predicted IZ greater than or equal to 23.50 mm. Pearson and Spearman correlation coefficients quantify concordance between the continuous model outputs. (**B**) Threshold-based overlap showing the SVC/MACCS-only, RegressionStack-only, and consensus-positive counts with true positive/false positive composition; summary annotations report selected counts and PPV for each full-model nomination set, the intersection, and the union. The union is presented as a capacity-dependent expansion set rather than as a default combined-model decision rule. (**C**) Active-hit overlap within the top 25 compounds prioritized by each model, showing active compounds shared by both ranked lists and those uniquely recovered by SVC/MACCS or RegressionStack. Model operating thresholds were selected from training out-of-fold predictions before evaluation on the locked external validation set.

Score concordance between SVC/MACCS calibrated probabilities and RegressionStack predicted-IZ values on the locked external validation set was moderate by Pearson correlation (r = 0.523) and weaker by rank correlation (Spearman ρ = 0.315) (Figure 10A; Table S11). At the predefined operating thresholds, the external compounds were partitioned into consensus-positive, SVC/MACCS-only, RegressionStack-only, and both-negative tiers.

The consensus-positive tier contained 35 compounds, comprising 27 TP and eight FP, with a PPV of 0.771. The SVC/MACCS-only tier contained 28 compounds, comprising 12 TP and 16 FP, with a PPV of 0.429. The RegressionStack-only tier contained 10 compounds, comprising four TP and six FP, with a PPV of 0.400. The union of both positive nomination sets contained 73 compounds, comprising 43 TP and 30 FP, with a PPV of 0.589. The both-negative tier contained 1057 compounds, including 44 measured actives, corresponding to an active fraction of 0.042 (Figure 10B; Table S12). Top-25 nomination analysis showed partial overlap between the two ranked outputs. SVC/MACCS recovered 16 measured actives among its top 25 compounds, whereas RegressionStack recovered 19. Nine active hits were shared between the two lists, while seven were unique to SVC/MACCS and 10 were unique to RegressionStack (Figure 10C; Table S11).

In the label-permutation nomination-null analysis, the consensus-positive tier recovered 27 actives compared with a permutation-null mean of 2.69, corresponding to 10.02-fold enrichment and an empirical *p* = 0.0001. The SVC/MACCS-only tier recovered 12 actives compared with a null mean of 2.14, corresponding to 5.57-fold enrichment and *p* = 0.0001. The RegressionStack-only tier recovered four actives compared with a null mean of 0.77, corresponding to 5.20-fold enrichment and *p* = 0.0055. The union recovered 43 actives compared with a null mean of 5.61, corresponding to 7.65-fold enrichment and *p* = 0.0001 (Figure S6; Table S12).

Threshold-margin analysis classified nine of the 28 SVC/MACCS-only nominations as near-boundary and 19 as higher-margin. Among the 10 RegressionStack-only nominations, five were near-boundary and five were higher-margin. Across both model-specific tiers, 14 of 38 nominations (36.8%) were classified as near-boundary, whereas 24 of 38 (63.2%) were classified as higher-margin (Figure S7; Table S12).

In the percentile-defined strong-disagreement subsets, one of the 37 compounds with high SVC/MACCS scores and low RegressionStack scores was active, whereas one of the 36 compounds with high RegressionStack scores and low SVC/MACCS scores was active.

### 2.9. Activity-Cliff Analysis near the IZ Boundary

An exploratory activity-cliff screen was performed to explore whether local structure–activity discontinuities might contribute to prediction difficulty near the activity threshold. This analysis identified 55 high-similarity Morgan fingerprint pairs with Tanimoto similarity ≥ 0.85 and absolute IZ differences ≥ 10 mm among 677 high-similarity pairs [36]; full pair-level activity-cliff data are included in the deposited data repository. SAR discontinuities near the activity boundary are a recognized source of QSAR prediction errors, and recent activity-cliff benchmarking has shown that such cases remain challenging even for modern QSAR models [37]. Such local discontinuities may contribute to the residuals observed in the 20–30 mm IZ range, where small structural changes can coincide with large differences in the measured inhibition-zone diameter. Therefore, future endpoint-matched antibacterial QSAR datasets should consider near-threshold SAR discontinuities, particularly around the IZ = 25 mm activity boundary.

## 3. Discussion

### 3.1. Matched-Data Evaluation of Complementary Model Roles

The principal methodological contribution of this study is a matched-data evaluation of two predefined QSAR roles using the same experimentally measured disk diffusion endpoint, compound sets, and preserved training and locked external validation partitions. SVC/MACCS was designed for precision-oriented active calling under the benchmark definition of measured IZ ≥ 25 mm, whereas RegressionStack used the continuous-IZ diameter as its training target and generated assay-referenced values for ranking and retrospective threshold analysis. The two outputs should therefore be interpreted as complementary computational views rather than as members of a statistically established performance hierarchy. Because the pipelines differ in endpoint formulation, model architecture, and molecular representation, the observed differences cannot be attributed solely to continuous versus binary endpoint treatment.

On the locked external benchmark, SVC/MACCS nominated 63 of 1130 compounds, including 39 measured actives, whereas RegressionStack produced capacity-dependent ranked and thresholded nomination sets. These results quantify retrospective PPV, enrichment, and nomination behavior under the evaluated data conditions. Whether comparable enrichment would be obtained when the workflows nominate previously unmeasured compounds remains a question for prospective experimental evaluation.

### 3.2. Performance Divergence, Error Structure, and Model Parsimony

The RegressionStack achieved higher point estimates than the SVC/MACCS model across several threshold-independent and early-recognition metrics (ROC-AUC, PR-AUC, EF@1%, PPV-25). However, paired permutation tests and bootstrap confidence intervals spanning zero revealed that these numerical differences were not statistically significant (ROC-AUC: *p* = 0.501; PR-AUC: *p* = 0.442). Accordingly, the observed numerical differences were not statistically resolved on the available external set. The distinct contribution of RegressionStack in this analysis is its assay-referenced output and associated retrospective threshold and nomination analyses, rather than demonstrated superiority in discrimination.

Architecturally, RegressionStack reduced the external-set MAE from 3.38 to 3.20 mm, corresponding to a 5.3% reduction relative to the strongest single specialist regressor, XGBoost/MACCS. This modest continuous-error reduction should be weighed against the additional complexity of the stacked architecture. However, the present analyses do not establish that a single regressor is an equivalent replacement for RegressionStack. The SVC/Morgan sensitivity analysis evaluated molecular representation within the binary classifier and does not address replacement of the stacked regression architecture.

Moreover, the prediction error increased across the measured IZ range: the overall MAE of 3.20 mm increased to 6.89 mm in the measured-active stratum (IZ ≥ 25 mm) and to 9.46 mm among high-IZ compounds (measured IZ ≥ 30 mm). All 30 high-IZ compounds were underpredicted. This systematic compression and the modest within-active Spearman correlation of 0.326 indicate that the continuous model supports coarse retrospective prioritization within the evaluated domain but not for resolving small activity differences among measured-active compounds. Because disk diffusion is a composite phenotype reflecting antibacterial effects and agar-diffusion behavior, neither model output should be interpreted as a direct measure of MIC, intrinsic molecular potency, or clinical efficacy [11,12,13].

### 3.3. Retrospective Hit-Tier Stratification and Nomination Complementarity

Nomination-level analysis provided the clearest evidence that the two workflows generated partially overlapping retrospective nomination sets. The consensus-positive tier contained 27 active compounds among 35 compounds, corresponding to a PPV of 0.771, whereas the SVC/MACCS-only and RegressionStack-only tiers yielded PPVs of 0.429 and 0.400, respectively.

Label-permutation analysis confirmed that all three tiers recovered significantly more actives than expected by chance (*p* < 0.05). Because 24 of the 38 model-specific discrepant nominations exceeded the predefined near-boundary criteria, disagreement was not confined to compounds immediately adjacent to the operating thresholds. This margin analysis does not identify the structural basis of disagreement or quantify prediction confidence. Extreme score discordance alone did not isolate an enriched subset. Under the evaluated retrospective conditions, the consensus-positive set represented the highest-PPV tier, whereas the model-specific sets represented lower-PPV but significantly enriched exploratory tiers. These designations describe retrospective nomination performance and do not establish prospective confidence or validation priority.

### 3.4. Robustness, Transferability, and ADs

The y-randomization null distributions were well below the observed values, supporting that the measured performance was unlikely to arise from randomized label or target associations under the implemented tests. In the separately assembled ChEMBL-derived transfer set, SVC/MACCS maintained threshold-independent discrimination, whereas RegressionStack retained early enrichment under greater chemical and assay-condition heterogeneity. Reconstructing the descriptor panel introduced numerical drift in 32 of 199 features but produced only small source-holdout prediction differences (mean absolute difference between reconstructed and saved predictions = 0.063 mm). However, the lower operating-point precision observed in the transfer set highlights that thresholds optimized on a single benchmark require dataset-specific empirical recalibration [38].

Active enrichment persisted under scaffold-grouped cross-validation (EF@1% of 6.52 ± 0.71 for SVC/MACCS; 8.28 ± 1.18 for RegressionStack). However, broader discrimination metrics declined relative to out-of-fold evaluation, indicating that both models rely on structurally related chemotypes in the training data. This is consistent with the external validation set, where 73.3% of the compounds shared a Bemis–Murcko scaffold with the training set, and all nominated hits fell within the Morgan/Tanimoto AD. The present evidence therefore supports retrospective prioritization primarily within the represented chemical domain; performance for unrelated or substantially novel scaffold spaces remains unestablished.

### 3.5. Mechanistic Plausibility and Activity Cliffs

Influential MACCS keys identified by feature attribution captured heteroatom-containing, ring-associated, polar/ionizable, and halogenated motifs. These features align with known Gram-negative permeation rules and agar diffusion physics, though they represent statistical associations rather than causal mechanisms of action [13,31,32,33,34,35]. Additionally, the identification of 55 highly similar compound pairs possessing localized IZ differences ≥ 10 mm highlights structural discontinuities within the dataset. These local activity cliffs are intrinsically difficult for both classification and regression topologies to resolve [36,37]. Reflecting either genuine biological shifts, diffusion anomalies, or experimental variance, these activity-cliff pairs may be suitable candidates for targeted experimental re-evaluation, but the current associative analyses do not establish their mechanistic basis.

### 3.6. Study Limitations and Future Directions

This study is a retrospective computational evaluation of published, experimentally measured disk diffusion records and a separately assembled ChEMBL-derived transfer set. No prospective experimental testing of compounds newly nominated by these workflows was performed; all reported nomination metrics were calculated retrospectively using compounds with existing experimentally measured IZ values. Accordingly, the observed PPV, enrichment, ranking, and nomination-overlap results quantify performance under the evaluated data conditions but do not establish prospective hit rates, reduced experimental workload, improved discovery yield, or pharmacological efficacy.

Several additional considerations bound the interpretation of the findings. First, the two pipelines differed not only in endpoint formulation but also in model architecture and molecular representation; consequently, the analysis does not isolate continuous versus binary endpoint treatment as the sole cause of their performance or nomination differences. Second, the locked external set functioned primarily as a defined-domain benchmark: 73.3% of the external compounds shared a Bemis–Murcko scaffold with the training set, and broader performance declined under repeated scaffold-grouped validation. Performance in substantially different or unrelated chemical space therefore remains unestablished. Third, RegressionStack systematically underpredicted compounds with high measured IZ values and showed modest ordering within the measured-active subset; predicted IZ should therefore be interpreted as a coarse prioritization coordinate rather than a millimeter-accurate estimate. Fourth, disk diffusion IZ is a composite phenotype influenced by antibacterial activity, agar diffusion, and physicochemical properties and is not a direct surrogate for MIC, intrinsic molecular potency, clinical susceptibility, or in vivo efficacy. Fifth, the ChEMBL-derived transfer set exhibited greater chemical and assay-condition heterogeneity, and the RegressionStack transfer analysis additionally relied on Mordred-reconstructed descriptors that showed numerical drift in 32 of 199 columns. Although prediction agreement on the source-paper external set remained close, the absolute RegressionStack transfer metrics should therefore be interpreted as descriptor-reconstruction and cross-dataset transfer sensitivity estimates rather than exact descriptor-transfer performance.

Future work should prospectively evaluate prespecified consensus-positive and model-specific nominations among compounds lacking pre-existing IZ measurements. Such evaluation should use standardized disk diffusion and MIC assays; include scaffold-novel compounds; prespecify the nomination rules, operating thresholds, and experimental budget; and report prospective hit rates and the experimental workload directly. This would determine whether the retrospective enrichment and nomination-tier structure observed here translate into improved compound selection under prospective screening conditions.

## 4. Materials and Methods

### 4.1. Study Design, Dataset, and Endpoint Definition

We conducted a retrospective ligand-based QSAR evaluation using experimentally measured disk diffusion IZ records originally curated by Bugeac et al. [20]. The primary objective was to compare the retrospective predictive and nomination behavior of two predefined modeling roles using the same compounds and preserved training and external validation partitions.

To address this objective, two modeling strategies were evaluated, each corresponding to a distinct screening method. The first approach employs a calibrated binary classifier designed for conservative active/inactive calling under the source benchmark activity definition. The second implemented a regression-first model in which IZ values were treated as a continuous variable, and binary thresholding was deferred until after prediction, allowing predicted-IZ values to be used directly as screening triage coordinates. This design follows QSAR best-practice principles regarding endpoint definition, external validation, AD, and alignment between model evaluation and intended practical use [6,7], which is consistent with recent benchmarking recommendations emphasizing that virtual screening evaluation should consider dataset quality, featurization, metrics, and data splits [8]. Because the intended application is virtual screening prioritization, evaluation is extended beyond conventional classification metrics to include nomination-set metrics, including PPV-k, top-k precision, and EFs, for imbalanced screening datasets [8,9,24].

**Figure 11:**
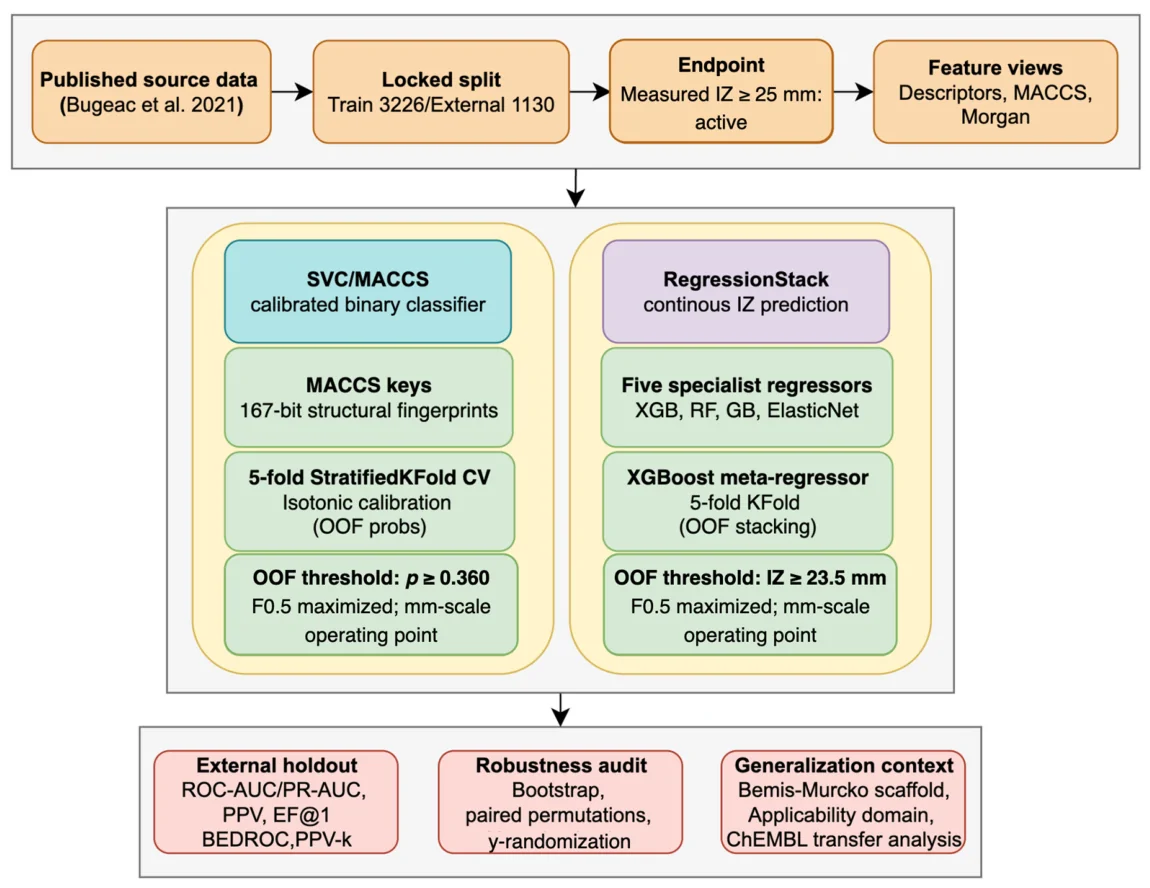
Study design and locked external validation workflow. The training (*n* = 3226; 240 actives) and external validation (*n* = 1130; 87 actives) sets of Bugeac et al. [20] were preserved without reassignment. Two model roles were prespecified: a calibrated SVC/MACCS binary classifier for conservative active calling (IZ ≥ 25 mm) and RegressionStack, a stacked ensemble trained to predict continuous IZ for ranking and threshold exploration. Decision thresholds were selected from training out-of-fold predictions only; the locked external set was used once for the final performance estimation. Robustness was assessed using bootstrap resampling, y-randomization, scaffold analysis, AD assessment, and ChEMBL-derived transfer.

To preserve direct comparability with the published benchmark and minimize external-set leakage, we retained the original training and external validation split. All model selection procedures, probability calibration, threshold optimization, and stacked-model meta-learning were performed exclusively using training data or training out-of-fold prediction. The external validation set remained completely locked during model development and was used only for the final performance estimation. The integrated workflow is shown in Figure 11.

The chemical structures and corresponding IZ measurements were obtained from the supplementary datasets provided by Bugeac et al. [20]. The source study curated disk diffusion assay data for *P. aeruginosa* from the ChEMBL bioactivity database [28] and reported fixed training and external validation partitions.

To ensure a direct comparison with the established benchmark, we used the original training and external validation partitions without any reassignment. The training set comprised 3226 compounds, including 240 active and 2986 inactive compounds, whereas the external validation set contained 1130 compounds, including 87 active and 1043 inactive ones. The active prevalence was 7.44% in the training set and 7.70% in the locked external validation set, representing the rare-positive structure typical of early-stage antibacterial virtual screening.

The primary experimental endpoint was the IZ measured in millimeters. To maintain direct comparability with the source benchmark, a binary activity label was established using the source’s 25 mm IZ cutoff. Compounds with an IZ ≥ 25 mm were classified as active (y = 1), while those with IZ < 25 mm were classified as inactive (y = 0).

This binary classification was applied to the SVC/MACCS classifier and all threshold-dependent performance evaluation. Conversely, continuous-IZ values were retained for the regression-first modeling pipeline to support compound ranking and assay-scale threshold exploration. The IZ ≥ 25 mm cutoff was used solely as the benchmark-specific activity definition and does not represent a Clinical and Laboratory Standards Institute (CLSI) susceptibility breakpoint.

### 4.2. Chemical Curation and Molecular Representation

We treated the source-paper training and external validation workbooks as curated benchmark datasets and maintained the original compound assignments between splits. Chemical validity, canonicalization, and duplicate verification were performed using the RDKit, an open-source cheminformatics toolkit [39]. The processed benchmark files included a training set and an external validation set, both consisting exclusively of valid, unique canonical Simplified Molecular Input Line Entry System (SMILES) strings with no missing IZ values or exact canonical duplicates.

To preserve the fidelity of the source benchmark data, no additional structural standardization procedures were applied, including salt stripping, tautomer enumeration, charge neutralization, or stereochemical collapse. A structure-format audit confirmed the absence of dot-disconnected salt or mixture records in either split. Stereochemical markers were identified in 206 training and 81 external structures, directional bond markers in 675 training and 250 external structures, and explicit bracket-charge annotations in 399 training and 137 external structures. No non-numeric or missing entries were detected across the 199 source descriptor columns in either of the splits. For the generated extended RDKit descriptors, columns with more than 20% missing values in the training set were removed, remaining missing or infinite values were median-imputed, and zero-variance columns were excluded. All filtering and imputation parameters were derived from the training set and applied to the external validation and transfer sets, where applicable, to prevent cross-set leakage.

Molecules were represented using fixed source-paper descriptors and molecular features derived from SMILES. The original workbooks provided 199 descriptor columns after excluding metadata and endpoint fields. Additional molecular features were generated using RDKit. Morgan fingerprints were calculated as 2048-bit circular fingerprints with a radius of 2 [40,41]. MACCS structural keys were generated as 167-position RDKit vectors, in which bit 0 was unused and positions 1–166 corresponded to the public MACCS key definitions described by Durant et al. [42]. These keys were selected because they provide sparse structural encoding with partial interpretability, using predefined substructure patterns. The molecular features were computed separately for each split of the dataset.

The software versions used for molecular curation, feature generation, and model fitting are listed in Table S13 in the Supporting Information. The original 199 source-paper descriptor columns were used directly for the primary benchmark analyses and were not re-generated. For the ChEMBL-derived RegressionStack transfer analysis, these descriptor columns were reconstructed using the exact descriptor name using Mordred. Because 32 of the 199 reconstructed descriptor columns showed numeric drift relative to the original source workbook, the RegressionStack transfer results were interpreted as a descriptor-reconstruction sensitivity analysis rather than as an exact descriptor transfer.

### 4.3. Model Training, Cross-Validation, and Hyperparameter Selection

All operating threshold selection and stacked-model meta-learning procedures used training data and training-derived out-of-fold predictions only; the locked external validation set was reserved for final performance estimation. For SVC/MACCS, the selected configuration was used to generate calibrated out-of-fold probabilities by five-fold shuffled stratified cross-validation with random seed 42. Within each outer-training partition, isotonic calibration used five-fold internal cross-validation. For RegressionStack, specialist-regressor and meta-regressor out-of-fold predictions were generated using five-fold shuffled cross-validation with random seed 42. The same fold definitions were used across specialist regressors, and stratification was not applied because the primary regression target was continuous IZ. A stage-by-stage summary is provided in Table S14.

To maintain methodological consistency, the same fold definitions were used across all the RegressionStack specialist regressors during the generation of the meta-regressor training data. This ensured that each meta-model training value was derived from specialist predictions for compounds not used during the fitting of the corresponding models. After out-of-fold predictions were generated, all final specialist models were refitted to the complete training set and applied once to the locked external validation set.

The detailed model configurations and hyperparameter settings are presented in Table S15. These included the SVC kernel and regularization parameter C; XGBoost parameters such as the number of estimators, maximum depth, learning rate, subsampling, column sampling, and regularization settings; random forest tree and feature sampling parameters; gradient-boosting parameters; ElasticNet regularization strength (α) and L1 mixing ratio (l1_ratio); calibration settings; and all associated random seeds.

SVC/MACCS hyperparameters were selected by three-fold stratified randomized search within the full training set. RegressionStack specialist and meta-regressor hyperparameters were selected using five-fold randomized searches, followed by five-fold out-of-fold prediction generation for stack construction and operating threshold selection. Because hyperparameter selection was not nested within the out-of-fold-generation folds, training-side out-of-fold estimates may be optimistic relative to a fully nested design. This limitation does not affect the independence of the locked external validation results because final configurations and operating thresholds were fixed before the models were refitted on the complete training set and applied to the external set.

### 4.4. Model Architectures

#### 4.4.1. Calibrated Binary SVC/MACCS Classifier

The primary binary classifier used an SVC model trained on MACCS keys and calibrated via isotonic regression. SVCs, a specific implementation of support vector machines (SVMs), are max-margin classifiers for two-group classification [43] and were implemented using the scikit-learn library [44]. Isotonic calibration was used to convert raw classifier scores into probability estimates; both isotonic and sigmoid calibration approaches have been characterized for supervised classifiers [45,46].

SVC/MACCS was selected as the primary binary classifier based on the training out-of-fold performance, MACCS-key interpretability, and calibrated probability outputs suitable for screening decision support. The final probability threshold was determined from training out-of-fold predictions by maximizing the F0.5 score, which prioritizes precision over recall. The selected operating threshold was a probability of ≥0.360.

#### 4.4.2. RegressionStack Continuous-IZ Model

RegressionStack was designed to predict continuous-IZ values and use these predictions as a threshold-flexible prioritization coordinate before applying any binary operating thresholds. This model follows the stacked generalization framework [47] and comprises five first-level specialist regressors: an XGBoost regressor trained on source-paper descriptors [48], a random forest regressor on Morgan fingerprints [49], a gradient boosting regressor on Morgan fingerprints [50], an XGBoost regressor on MACCS keys, and an ElasticNet regressor on source-paper descriptors [51]. Specialist out-of-fold predictions were then combined using an XGBoost meta-regressor to generate the final predicted-IZ output.

The primary RegressionStack operating threshold of predicted IZ ≥ 23.50 mm was selected from training out-of-fold predicted values by maximizing F0.5 against the binary IZ ≥ 25 mm activity label. This threshold served as a model operating point, and the ground-truth activity definition remained measured IZ ≥ 25 mm throughout the study period.

#### 4.4.3. SVC/Morgan Descriptor-Representation Sensitivity Analysis

To assess whether the primary binary SVC/MACCS comparator was disadvantaged by the use of relatively simple MACCS structural keys, we performed an additional descriptor-representation sensitivity analysis using Morgan fingerprints. The SVC/Morgan model used 2048-bit Morgan fingerprints with radius 2 and the same binary activity definition as the primary classifier, measured IZ greater than or equal to 25 mm. Model selection, isotonic calibration, and operating threshold selection were performed using training set data and training out-of-fold predictions. The locked external validation set was used only after the SVC/Morgan pipeline and the operating threshold were fixed. This analysis was used as a sensitivity comparison for descriptor representation and was not used to redefine primary binary comparators.

### 4.5. Performance Metrics, Residual Diagnostics, and Calibration

The model performance on the locked external validation set was evaluated using continuous-target metrics, threshold-dependent classification metrics, threshold-independent ranking metrics, screening-oriented nomination metrics, and probability-calibration metrics, where applicable.

For continuous-IZ prediction, we calculated the RMSE, MAE, R^2^, Pearson’s correlation, Spearman’s rank correlation, and Lin’s CCC. The CCC was included because it jointly captures the correlation and systematic bias between the measured and predicted values [52,53].

To evaluate whether the RegressionStack error varied across the measured IZ range, residual diagnostics were performed on a locked external validation set. Residuals were defined as the predicted IZ minus the measured IZ; thus, negative residuals indicated underprediction. Error metrics were summarized across measured IZ bins of less than 15 mm, 15–19.9 mm, 20–24.9 mm, 25–29.9 mm, and greater than or equal to 30 mm. The measured IZ greater than or equal to 25 mm subset was additionally evaluated as the active stratum, and the measured IZ greater than or equal to 30 mm subset was evaluated as the high-IZ stratum. Pearson and Spearman correlations were also calculated within the active stratum to assess whether the predicted IZ retained within-active ordering information despite the increased absolute error.

Because the residual diagnostics indicated systematic high-IZ underprediction, a training-only monotonic calibration sensitivity analysis was performed for the RegressionStack predicted-IZ values. Four candidate mappings were evaluated using training out-of-fold prediction pairs only: identity, ordinary least squares linear calibration, robust Huber linear calibration, and isotonic calibration. Calibration selection used repeated internal validation across the same five measured IZ bins, with the macro-averaged bin-wise MAE as the primary criterion and the overall MAE retained as a safety check. The locked external validation set was not used for calibration selection; the selected mapping was applied to the external set only after the calibration rule had been fixed. This analysis was used to test whether high-IZ underprediction reflected a correctable monotonic scale-calibration artifact rather than defining a new primary model.

Threshold-dependent binary classification performance was evaluated using the PPV, sensitivity, specificity, balanced accuracy, F0.5, F1, and Matthews correlation coefficient (MCC). The MCC was retained as a threshold-dependent performance metric.

For threshold-independent ranking, we assessed the ROC-AUC and PR-AUC. The PR-AUC was emphasized because active compounds are rare, and this metric is more informative than the ROC-AUC under a strong class imbalance [22,23]. Screening-oriented nomination performance was evaluated using PPV-k, top-k active recovery, and enrichment over random expectation at fixed hit-list sizes of k = 25, 50, and 100. These metrics are analogous to the PPV-N criteria proposed for virtual screening workflows with limited experimental capacity [8] and are consistent with broader recommendations for evaluating virtual screening methods [54]. Early recognition was additionally summarized using an EF@1% and BEDROC with α = 20.0 [24]. The α = 20.0 parameter was selected because it corresponds to exponential decay weighting that concentrates scoring emphasis on approximately the top 8% of the ranked list, a stringency level appropriate for virtual screening settings in which only a small fraction of nominated compounds can be advanced to experimental testing [24]. BEDROC was treated as a secondary metric because it depends on the user-defined α parameter and is less directly interpretable than nomination-set precision [8].

Probability calibration was evaluated for SVC/MACCS using the Brier score, log loss, and ECE computed over ten fixed-width probability bins spanning the probability interval from 0 to 1 [38,45,46]. Probability-calibration metrics were not applied to RegressionStack because the predicted-IZ values were not probabilities.

The metric interpretation was aligned with the screening task. PPV quantified hit-list purity among nominated compounds; sensitivity quantified the fraction of true actives recovered; specificity quantified rejection of inactive compounds; balanced accuracy averaged sensitivity and specificity under class imbalance; F0.5 summarized a precision-weighted threshold tradeoff; F1 summarized the equal precision–recall tradeoff; and MCC summarized binary prediction agreement while accounting for all four confusion-matrix cells. For continuous-IZ prediction, MAE and RMSE quantified the prediction error in millimeters, R^2^ quantified the explained variance, Pearson r and Spearman ρ quantified linear and rank agreement, and CCC quantified concordance while penalizing systematic bias. For ranking, ROC-AUC measures the global ordering of actives above inactives, whereas PR-AUC, PPV-k, EFs, and BEDROC emphasize active recovery in rare-positive screening settings. The Brier score, log loss, and ECE assessed whether the calibrated probability outputs matched the observed active frequencies, which is important for threshold-based decision support.

### 4.6. Statistical Validation and Model-Difference Uncertainty

Uncertainty in external validation performance estimates was assessed using bootstrap resampling with 1000 resamples of the locked external validation set [25]. Model comparison was first conducted using paired permutation tests for the ROC-AUC and PR-AUC. Because paired permutation *p*-values alone do not quantify the magnitude or uncertainty of model differences, paired bootstrap confidence intervals were additionally calculated for RegressionStack minus SVC/MACCS performance differences using 10,000 paired resamples of the locked external validation set. In each bootstrap iteration, the same resampled compound indices were applied to both models, preserving the paired structure of the comparison. Confidence intervals were calculated for ROC-AUC, PR-AUC, EF@1%, PPV, balanced accuracy, and top-k PPV. These paired bootstrap intervals were used to assess whether the observed point estimate differences supported model-wide superiority or were more appropriately interpreted as uncertain descriptive differences.

To test whether the observed performance exceeded the randomized label or randomized target null distributions, y-randomization was performed as a standard QSAR null test [26,27]. For SVC/MACCS, the binary training labels were randomly permuted before retraining. For RegressionStack, continuous training IZ values were permuted, followed by retraining of all specialist regressors and the meta-regressor under fixed hyperparameters. Each experiment comprised 100 permutations, with empirical *p*-values calculated using a plus-one correction method. Therefore, *p* = 0.0099 represents the minimum attainable empirical *p*-value for 100 permutations and indicates that no randomized model matched or exceeded the corresponding observed metric. These tests were interpreted as evidence against chance correlation, rather than as high-resolution significance estimates.

For the descriptor-representation sensitivity analysis, the same paired bootstrap procedure was applied to differences defined as SVC/Morgan minus SVC/MACCS using 10,000 paired resamples of the locked external validation set. Confidence intervals were calculated for PPV, ROC-AUC, PR-AUC, EF@1%, and PPV-25 using identical resampled compound indices for both representations. Balanced accuracy, PPV-50, and PPV-100 were reported descriptively because paired bootstrap confidence intervals were not calculated for these metrics.

### 4.7. Scaffold-Grouped Validation and Applicability Domain Analysis

The chemical space overlap between the training and external validation sets was assessed using canonical SMILES and Bemis–Murcko scaffolds [55]. Scaffold and AD analyses were included because nominal external sets often assess interpolation within the represented chemical series rather than extrapolation to novel chemotypes [6,7,56]. To evaluate the model performance under stricter scaffold separation, repeated scaffold-grouped validation was performed using Bemis–Murcko scaffold groups generated from canonical SMILES. Acyclic compounds were assigned fallback scaffold groups based on their achiral canonical SMILES to avoid the collapse of all acyclic molecules into a single artificial group. Scaffold-grouped validation was performed using five folds and three repeated group-stratified splits with random seeds 42, 101, and 202, while ensuring that compounds sharing the same scaffold group did not appear in both training and held-out folds.

For SVC/MACCS, the primary binary classification pipeline was re-evaluated under scaffold-grouped validation using the same modeling framework, isotonic calibration strategy, and precision-weighted F0.5 threshold-selection criterion. For the RegressionStack, a near-exact fixed-architecture scaffold-grouped sensitivity analysis was performed. The original five-specialist stacked architecture and XGBoost meta-regressor were retained and retrained within each scaffold-grouped outer fold, but the manuscript hyperparameter settings were kept fixed rather than repeating the full randomized hyperparameter-search procedure in every fold. Fold-specific operating thresholds were selected using only outer-training predictions. These scaffold-grouped analyses were used to assess whether active enrichment survived scaffold separation, not to replace the locked external validation benchmarks.

The chemical space structure was visualized using principal component analysis (PCA) of the molecular fingerprints. The AD was defined by the nearest-neighbor Tanimoto similarity to the training set, computed using 2048-bit Morgan fingerprints with a radius of 2. External compounds with a maximum training-set Tanimoto similarity ≥ 0.474 were classified as being within the domain. Following nearest-neighbor AD concepts [56], this empirical threshold was set to the 5th percentile of leave-one-out nearest-neighbor Tanimoto similarities in the training set. Coverage, predicted-positive counts, and domain-stratified performance metrics were calculated separately for external compounds classified as inside and outside the AD. Outside-domain PPV was interpreted descriptively when the number of predicted positives was small.

### 4.8. ChEMBL-Derived Transfer and Descriptor Reconstruction

To evaluate cross-dataset transfer beyond the source-paper holdout, a ChEMBL-derived disk diffusion/IZ endpoint set was assembled [28]. Records were retained only if they included usable SMILES strings, a disk diffusion or IZ endpoint annotation, an equality relation, and an IZ value interpretable in millimeters. Canonical SMILES strings were generated using RDKit, and any exact canonical SMILES overlap with source-paper compounds was removed before evaluation. The final transfer set comprised 1458 compounds with 75 actives, corresponding to 5.1% active prevalence, and exhibited zero exact canonical SMILES overlap with the original source-paper compounds. Parent- and scaffold-level overlaps were subsequently computed as sensitivity metadata.

For evaluation, SVC/MACCS was applied directly to the ChEMBL-derived transfer set without model refitting. RegressionStack was also applied without refitting after reconstructing the 199 source-paper descriptor columns by exact name using Mordred [29]. Because 32 of the 199 reconstructed descriptor columns exhibited numerical drift relative to the original source workbook, the ChEMBL-derived RegressionStack results were interpreted as a descriptor-reconstruction and cross-dataset transfer sensitivity analysis rather than as exact descriptor-transfer performance. These transfer results were not used to revise the primary conclusions from the locked source-paper holdout. To quantify the practical impact of descriptor drift, reconstructed and originally saved RegressionStack predictions were compared systematically on the locked source-paper external set before transfer evaluation; the corresponding source files and agreement summary are included in the deposited data repository.

### 4.9. Feature Interpretation

Feature interpretation focused on the MACCS-based classifier because MACCS keys can be mapped to predefined structural motifs with established definitions [42]. SHapley Additive exPlanations (SHAP) analysis [30] was applied to identify the influential MACCS keys, which were interpreted using predefined MACCS key definitions, where available [42]. Feature attribution was interpreted as associative rather than mechanistic because disk diffusion IZ values reflect both the antibacterial effect and physicochemical determinants of agar diffusion [11,13].

### 4.10. Nomination Overlap, Label-Permutation Null, and Tiered Complementarity Analysis

Nomination-level overlap between SVC/MACCS and RegressionStack was assessed using the locked external validation set with saved final model predictions without model refitting. Score concordance was quantified using Pearson and Spearman’s correlation coefficients. Fixed-threshold overlap was evaluated using the operating thresholds selected from training out-of-fold predictions: SVC/MACCS probability ≥ 0.360 and RegressionStack predicted IZ ≥ 23.50 mm. The nominations were grouped into four tiers: consensus-positive, SVC/MACCS-only, RegressionStack-only, and both-negative compounds. For each tier, the selected compound counts, TP, FP, PPV, and sensitivity were calculated. The union of model-selected compounds was evaluated as an exploratory expansion set rather than as a default-combined model decision rule.

A label-permutation nomination-null analysis was performed by holding model predictions, thresholds, rankings, and nomination memberships fixed while randomly permuting external active/inactive labels 10,000 times. Empirical *p*-values were calculated using a plus-one correction method. This analysis tested fixed-nomination enrichment relative to random label assignment, not retraining model stability.

Threshold-margin analysis was performed for the model-specific nominations. The SVC/MACCS margin was defined as the calibrated probability minus 0.360, and the RegressionStack margin was defined as the predicted IZ minus 23.50 mm. Two descriptive near-boundary rules were applied. The absolute cutoffs were margins from 0 to less than 0.05 probability units for SVC/MACCS and from 0 to less than 1.0 mm for RegressionStack. The model-specific relative cutoffs were defined by the first quartile (Q1) of the positive-margin distributions: 0.0587599 probability units for SVC/MACCS and 0.670473 mm for RegressionStack. A model-specific nomination was classified as near-boundary if it satisfied either the absolute cutoff or the relative-Q1 cutoff; all remaining nominations were classified as higher-margin. Strong-disagreement subsets were defined using within-model percentile ranks. High-SVC/low-RegressionStack compounds had SVC/MACCS percentile ranks ≥ 0.75 and RegressionStack percentile ranks ≤ 0.25; high-RegressionStack/low-SVC compounds met the converse criteria.

## 5. Conclusions

This retrospective QSAR evaluation compared two predefined modeling roles using the same experimentally measured *P. aeruginosa* disk diffusion records and preserved training and locked external validation partitions. SVC/MACCS supported precision-oriented active calling under the benchmark definition of measured IZ ≥ 25 mm, whereas RegressionStack used continuous-IZ diameter as its training target and generated predicted-IZ values for coarse compound prioritization and retrospective threshold exploration. Although RegressionStack produced numerically higher values for several ranking metrics, the between-workflow differences were not statistically resolved. The results therefore support a complementary, rather than hierarchical, interpretation of the two workflows. This complementarity was also reflected in their partially overlapping nomination sets, with the consensus-positive set containing 27 measured actives among 35 nominations and showing the highest PPV among the evaluated retrospective tiers (0.771). The y-randomization analyses supported the presence of a non-random structure–activity signal, while locked external validation, uncertainty analysis, scaffold-grouped evaluation, cross-dataset transfer sensitivity analysis, and nomination-level label-permutation testing characterized the robustness, uncertainty, and domain dependence of the retrospective prioritization results. Because the workflows differed in endpoint formulation, model architecture, and molecular representation, their performance and nomination differences cannot be attributed solely to continuous versus binary endpoint treatment. Furthermore, the systematic underprediction of compounds with high measured IZ values and modest ordering within the measured-active subset indicate that predicted IZ should be interpreted as a coarse prioritization coordinate rather than a millimeter-accurate estimate or a surrogate for intrinsic antibacterial potency, clinical susceptibility, or therapeutic efficacy. No prospective experimental validation of model-nominated compounds was performed. Consequently, whether the observed retrospective enrichment translates into improved prospective hit discovery or reduced experimental workload remains to be established through prospective testing.

## Data Availability

The source benchmark data were obtained from the supplementary files of Bugeac et al. [20]. The ChEMBL-derived transfer set was assembled from publicly available ChEMBL records [28] using the filtering criteria described in the Section 4. Processed datasets, model development and evaluation code, validation predictions, metric summaries, and deposited support files for the activity cliff and descriptor-reconstruction analyses are available at https://github.com/SukritKashyap/pa-aeruginosa-iz-qsar-model (accessed on 14 July 2026).

## Supplementary Materials

The following supporting information can be downloaded at: https://www.mdpi.com/article/10.3390/ph19081173/s1, Figure S1: Early-recognition performance on the locked external validation set; Figure S2: Probability-calibration analysis for SVC/MACCS; Figure S3: Sensitivity of calibrated SVC performance to molecular representation on the locked external validation set; Figure S4: Training-only calibration sensitivity analysis for RegressionStack-predicted inhibition-zone values; Figure S5: Relative change in model performance under scaffold-grouped validation; Figure S6: Fixed-nomination label-permutation analysis of model-overlap sets on the locked external validation set; Figure S7: Threshold-margin analysis of model-specific nominations on the locked external validation set; Table S1: Locked-external sensitivity comparison of SVC/MACCS and the training-selected SVC/Morgan representation; Table S2: Overall RegressionStack prediction diagnostics and central-tendency baseline comparison on the locked external validation set; Table S3: RegressionStack residual diagnostics stratified by measured inhibition-zone diameter on the locked external validation set; Table S4: Performance comparison of the RegressionStack specialist regressors and final stacked model on the locked external validation set; Table S5: Top-k hit-list recovery and enrichment; Table S6: Descriptor-reconstruction and prediction-agreement sensitivity analysis on the source-paper external validation set; Table S7: Conventional out-of-fold and repeated scaffold-grouped validation performance for SVC/MACCS and RegressionStack; Table S8: Scaffold-stratified external validation for SVC/MACCS and RegressionStack; Table S9: Applicability-domain analysis; Table S10: SHAP-ranked MACCS feature attribution for the SVC/MACCS classifier; Table S11: Nomination-overlap and operational complementarity summary; Table S12: Tiered nomination, label-permutation null, and model-specific threshold-margin summary on the locked external validation set; Table S13: Software versions and computational environment used for the primary analyses, descriptor reconstruction, and reviewer-requested sensitivity analyses; Table S14: Cross-validation and training-side model-selection design; Table S15: Hyperparameter configurations.

## Abbreviations

The following abbreviations are used in this manuscript:

AD	Applicability domain
AMR	Antimicrobial resistance
BEDROC	Boltzmann-enhanced discrimination of receiver operating characteristic
CCC	Concordance correlation coefficient
CLSI	Clinical and Laboratory Standards Institute
ECE	Expected calibration error
EF	Enrichment factor
EF@1%	Enrichment factor at 1% of the ranked list
F0.5	F-score with β = 0.5, precision-weighted F-measure
F1	F-score with equal weighting of precision and recall
FP	False positive
IZ	Inhibition-zone
MACCS	Molecular ACCess System structural keys
MAE	Mean absolute error
MCC	Matthews correlation coefficient
MIC	Minimum inhibitory concentration
PCA	Principal component analysis
PPV	Positive predictive value
PPV-k	Positive predictive value at fixed top-k list size
PPV-25	Positive predictive value among the top 25 ranked compounds
PR	Precision–recall
PR-AUC	Precision–recall area under the curve
QSAR	Quantitative structure–activity relationship
R^2^	Coefficient of determination
RDKit	Open-source cheminformatics toolkit
RMSE	Root mean square error
ROC	Receiver operating characteristic
ROC-AUC	Receiver operating characteristic area under the curve
SAR	Structure–activity relationship
SHAP	SHapley Additive exPlanations
SMILES	Simplified Molecular Input Line Entry System
SVC	Support vector classifier
SVM	Support vector machine
TP	True positive
XGBoost	Extreme gradient boosting
