# Supplementary material for: Continuous Inhibition-Zone Modeling and Binary Classification for Pseudomonas aeruginosa Hit Prioritization: A Retrospective QSAR Evaluation
Source: Pharmaceuticals (Basel). 2026 Jul 27;19(8):1173. doi: 10.3390/ph19081173 (PMC13516549; doi:10.3390/ph19081173)
Supplement: Supplementary file 1 [file pharmaceuticals-19-01173-s001.zip › pharmaceuticals-4397344-supplementary.pdf]

Supporting Information

Supplementary Figures

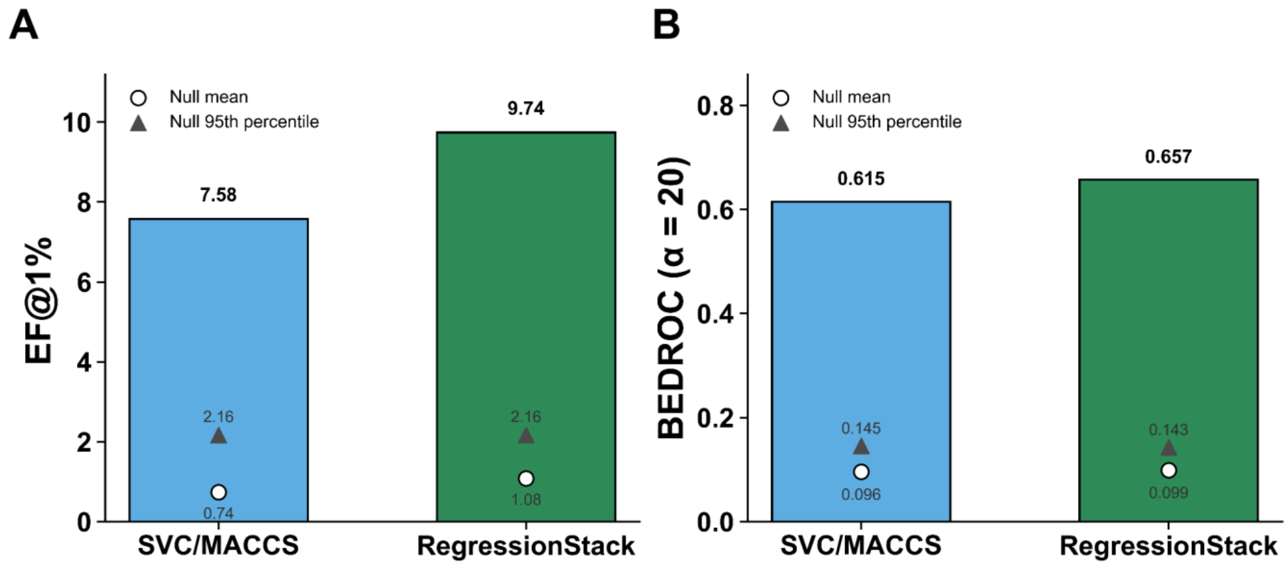

Figure S1. Early-recognition performance on the locked external validation set. (A) EF@1% for SVC/MACCS and RegressionStack. (B) BEDROC with $\alpha = 20.0$. Bars show observed model performance; open circles show the 100-permutation null mean; triangles show the null 95th percentile.

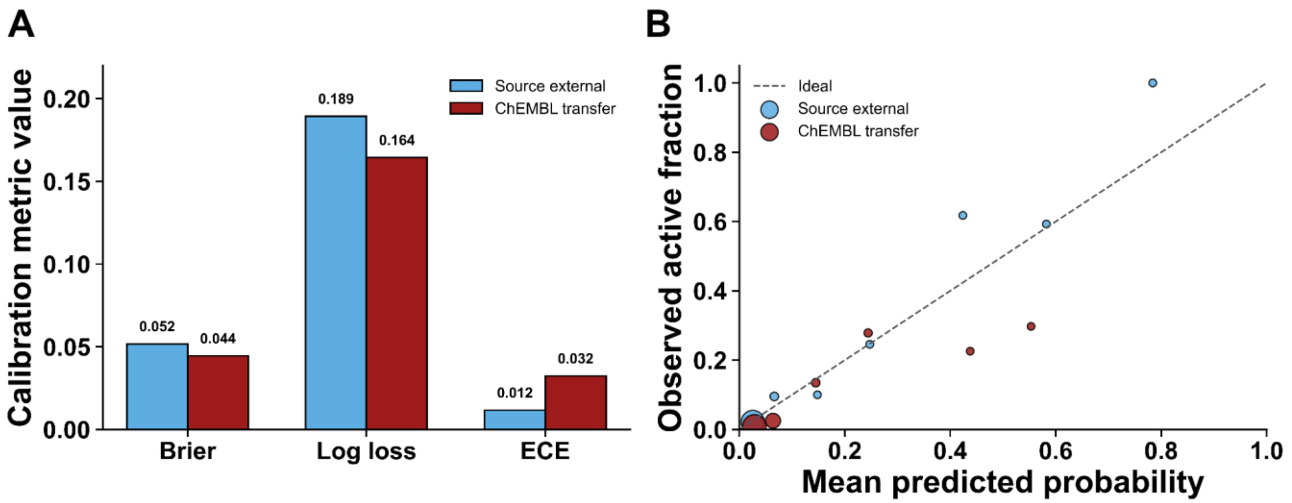

Figure S2. Probability-calibration analysis for SVC/MACCS. (A) Brier score, log loss, and 10-bin ECE for the source-paper external validation set and ChEMBL-derived transfer set. (B) Reliability plot showing mean predicted probability versus observed active fraction across fixed probability bins; marker size is proportional to bin count.

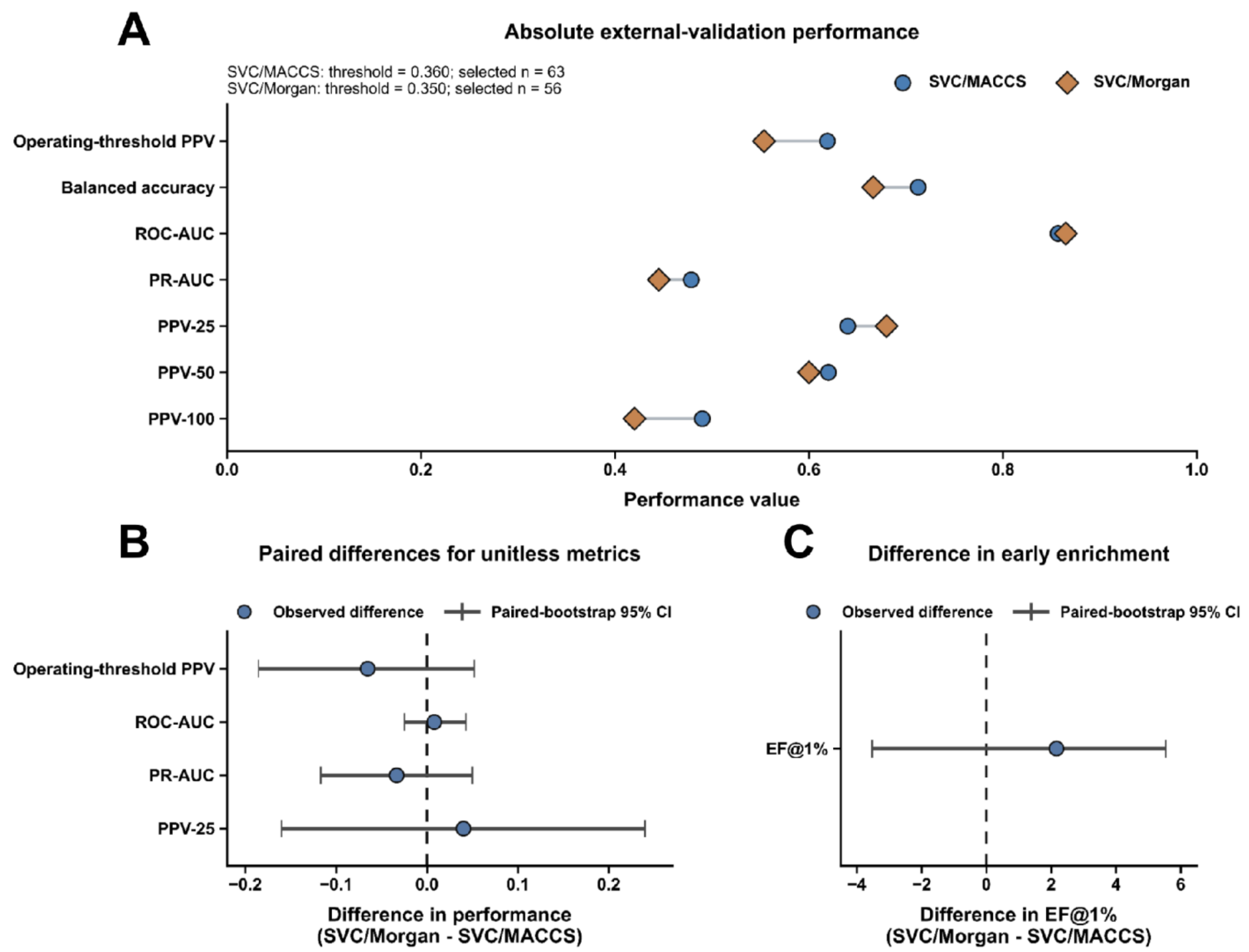

Figure S3. Sensitivity of calibrated SVC performance to molecular representation on the locked external validation set. (A) Absolute performance of SVC/MACCS and SVC/Morgan across threshold-dependent, ranking, and top-k metrics. Training-derived thresholds were 0.360 and 0.350, selecting 63 and 56 compounds, respectively. (B) Paired differences for unitless metrics and (C) for EF@1%, calculated as SVC/Morgan minus SVC/MACCS. Symbols denote point estimates, horizontal bars denote percentile-based 95% confidence intervals from 10,000 paired bootstrap resamples, and dashed vertical lines denote zero difference.

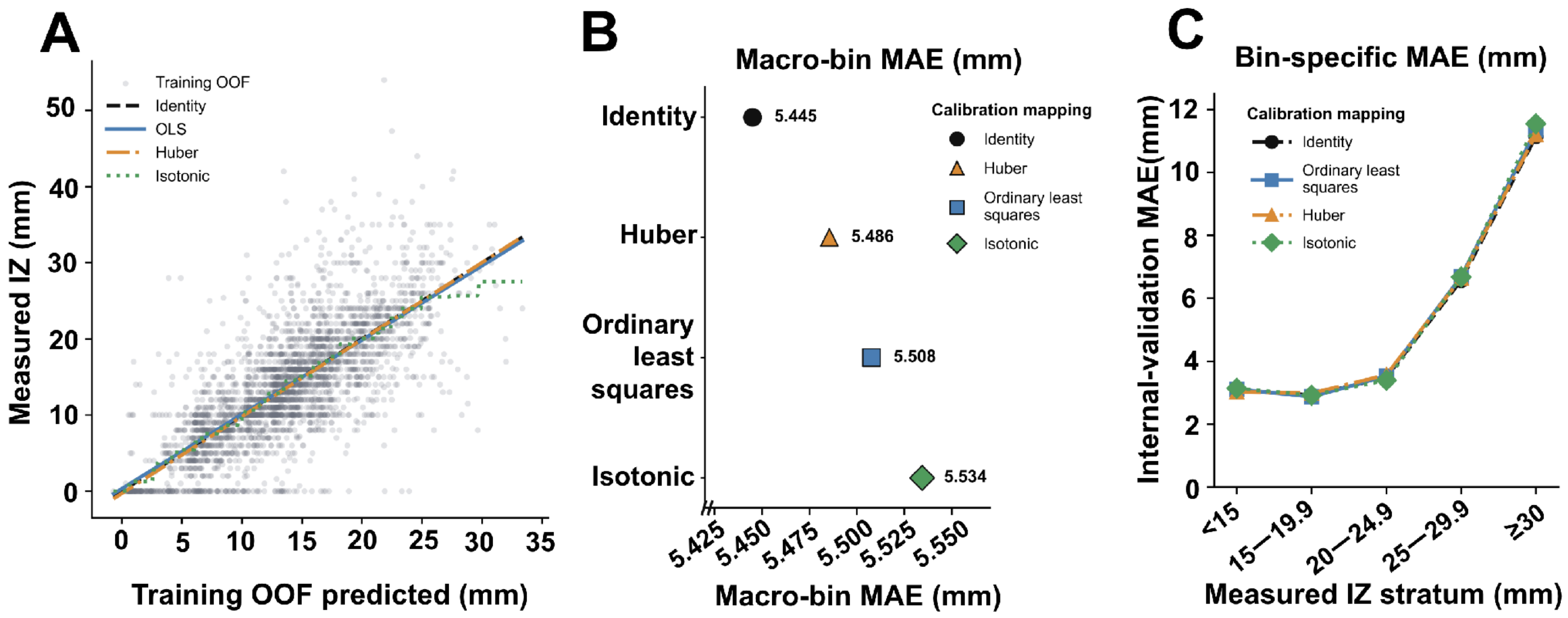

Figure S4. Training-only calibration sensitivity analysis for RegressionStack-predicted inhibition-zone values. (A) Relationship between training out-of-fold (OOF) predictions and measured inhibition-zone (IZ) diameters, with identity, ordinary least-squares (OLS), Huber, and isotonic calibration mappings. Grey points represent individual OOF prediction pairs. (B) Macro-bin mean absolute error (MAE, mm) across the predefined measured-IZ strata for each calibration mapping. (C) Bin-specific MAE (mm) within each measured-IZ stratum. Calibration selection was based exclusively on training OOF predictions; the identity mapping yielded the lowest macro-bin MAE and was therefore retained without reference to the locked external validation set.

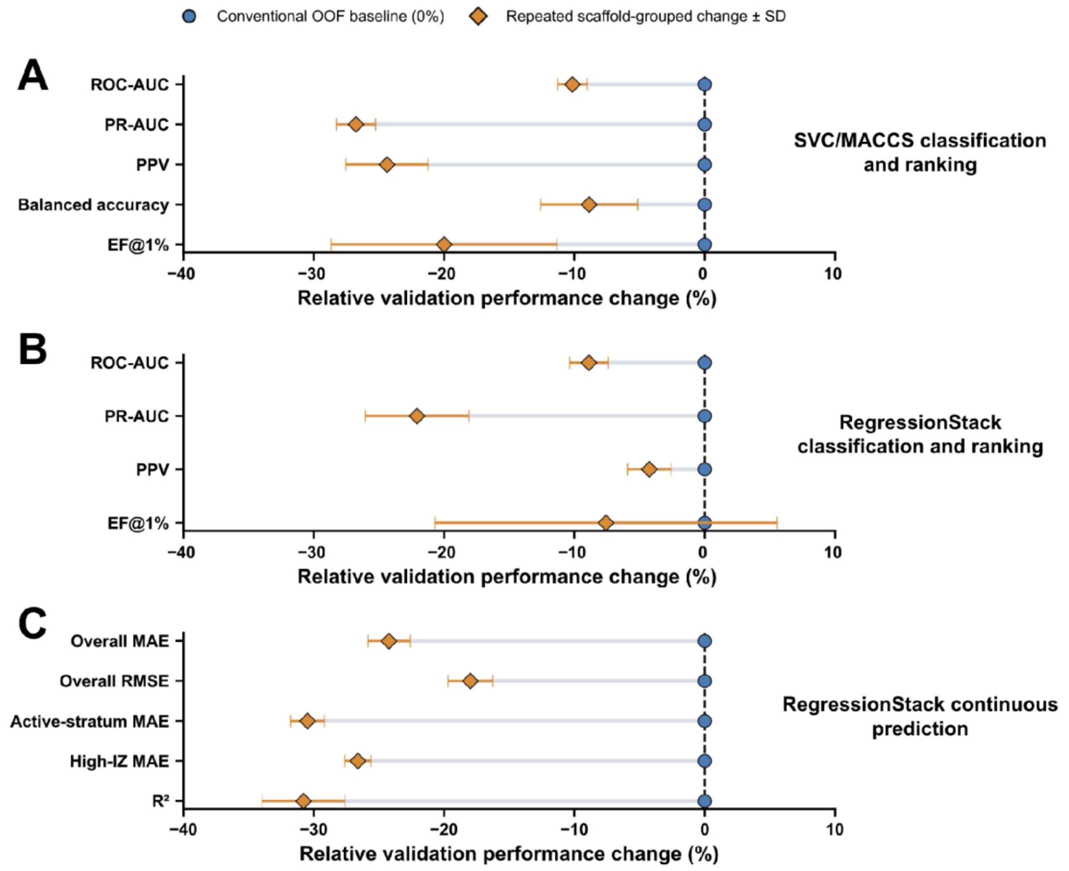

Figure S5. Relative change in model performance under scaffold-grouped validation. Conventional out-of-fold (OOF) performance was set as the 0% reference (blue circles). Orange diamonds show the mean relative change across three repeated five-fold Bemis–Murcko scaffold-grouped validation runs, with horizontal error bars indicating the relative standard deviation (SD) across seeds 42, 101, and 202. (A) SVC/MACCS discrimination, threshold-dependent classification, and early-enrichment metrics. (B) RegressionStack discrimination and early-enrichment metrics based on predicted inhibition zone (IZ) rankings, together with PPV calculated at fold-specific operating thresholds selected using only the corresponding outer-training partition. (C) RegressionStack continuous-prediction metrics, including overall MAE and RMSE, active-stratum MAE (measured IZ ≥ 25 mm), high-IZ MAE (measured IZ ≥ 30 mm), and R^2. Relative change was calculated as 100 × (scaffold-grouped mean − conventional OOF)/conventional OOF. For lower-is-better error metrics, the sign was reversed so that negative values consistently indicate performance degradation and positive values indicate improvement.

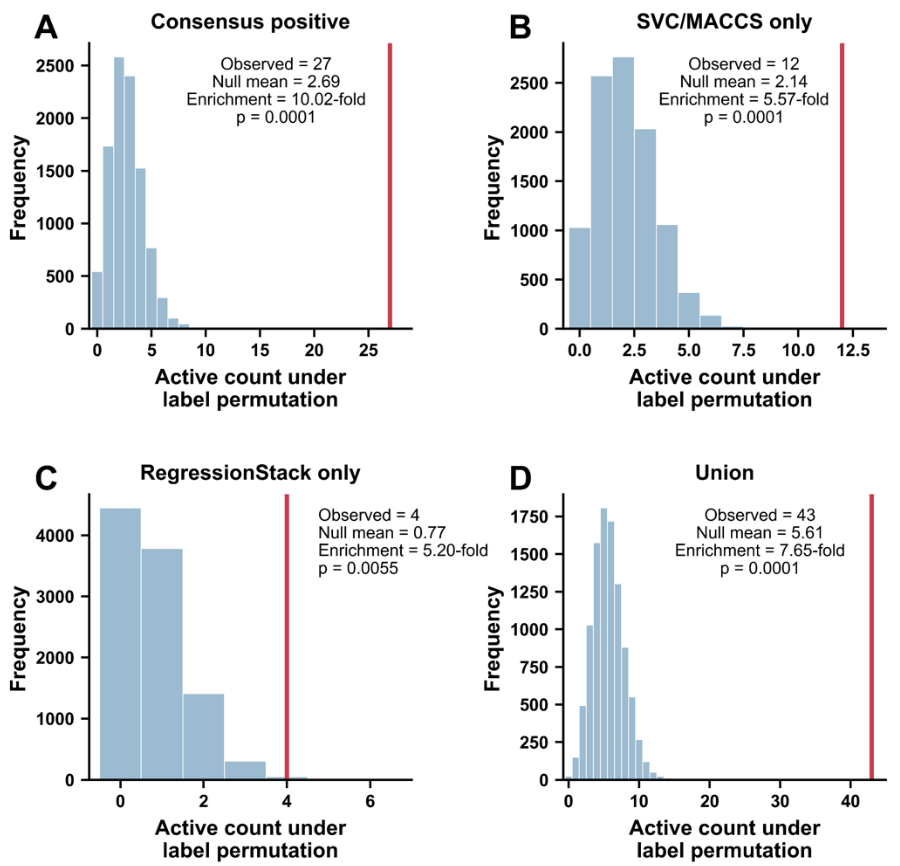

Figure S6. Fixed-nomination label-permutation analysis of model-overlap sets on the locked external validation set. Nomination sets were defined using the prespecified thresholds of SVC/MACCS probability ≥ 0.360 and RegressionStack predicted inhibition-zone value ≥ 23.50 mm. Histograms show active counts obtained from 10,000 permutations of the measured binary labels while preserving the total number of actives (n = 87); model predictions, thresholds, rankings, and set memberships were held fixed. Panels show (A) consensus-positive, (B) SVC/MACCS-only, (C) RegressionStack-only, and (D) union nomination sets. Red vertical lines indicate observed active counts. Annotations report the observed count, permutation-null mean, fold enrichment, and one-sided empirical p-value with plus-one correction. This analysis assesses enrichment of fixed nomination sets under random label assignment, not nomination stability under model retraining or statistical independence between models.

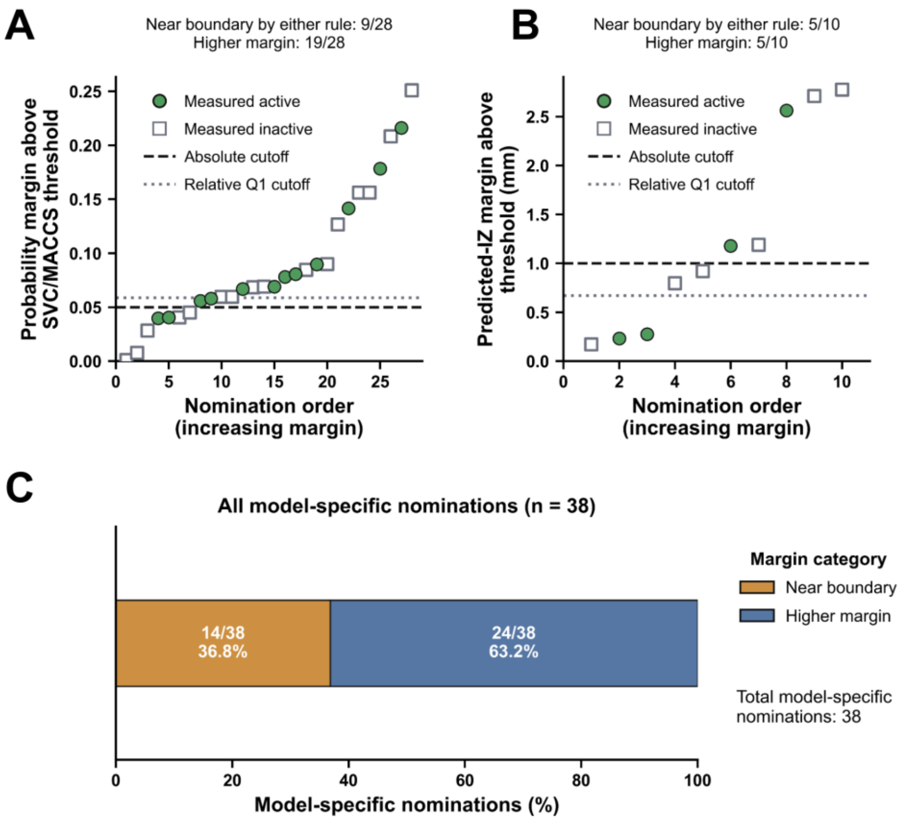

Figure S7. Threshold-margin analysis of model-specific nominations on the locked external validation set. (A) SVC/MACCS-only nominations (n = 28) ordered by increasing probability margin above the 0.360 operating threshold. (B) RegressionStack-only nominations (n = 10) ordered by increasing predicted inhibition-zone (IZ) margin above the 23.50 mm operating threshold. Filled circles and open squares denote measured active (IZ ≥ 25 mm) and inactive compounds, respectively. Dashed lines indicate absolute near-boundary cutoffs of 0.05 probability units and 1.0 mm, whereas dotted lines indicate the corresponding lowest-quartile positive-margin cutoffs of 0.0588 and 0.670 mm, respectively. Nominations meeting either criterion were classified as near-boundary. (C) Pooled distribution of the 38 model-specific nominations according to margin category. Threshold margins were evaluated descriptively and do not quantify prediction confidence or nomination stability.

Supplementary Tables

Table S1. Locked-external sensitivity comparison of SVC/MACCS and the training-selected SVC/Morgan representation. **Note:** Differences were calculated as SVC/Morgan minus SVC/MACCS. Percentile-based 95% confidence intervals were obtained from 10,000 paired bootstrap resamples of the 1,130-compound locked external validation set. EF@1% was calculated using the top 12 ranked compounds. SVC/Morgan and RegressionStack each recovered nine measured actives, with four overlapping compounds, three of which were active; therefore, their identical EF@1% estimates were coincidental. Confidence intervals were not calculated for the operating threshold, selected-compound count, balanced accuracy, PPV-50, or PPV-100.

Metric	SVC/MACCS	SVC/Morgan	Difference (SVC/Morgan − SVC/MACCS)	Paired-bootstrap 95% CI	CI includes zero
Operating threshold	0.360	0.350	−0.010	Not computed	—
Selected compounds	63	56	−7	Not computed	—
PPV	0.619	0.554	−0.065	−0.185 to 0.052	Yes
Balanced accuracy	0.713	0.666	−0.046	Not computed	—
ROC-AUC	0.857	0.865	0.008	−0.025 to 0.043	Yes
PR-AUC	0.479	0.445	−0.034	−0.117 to 0.050	Yes
EF@1%	7.58	9.74	2.165	−3.531 to 5.539	Yes
PPV-25	0.640	0.680	0.040	−0.160 to 0.240	Yes
PPV-50	0.620	0.600	−0.020	Not computed	—
PPV-100	0.490	0.420	−0.070	Not computed	—

Abbreviations: CI, confidence interval; EF@1%, enrichment factor at 1% of the ranked list; MACCS, Molecular ACCess System structural keys; PPV, positive predictive value; PPV-25, PPV-50, and PPV-100, positive predictive value among the top 25, 50, and 100 ranked compounds, respectively; PR-AUC, precision–recall area under the curve; ROC-AUC, receiver operating characteristic area under the curve; SVC, support vector classifier.

Table S2. Overall RegressionStack prediction diagnostics and central-tendency baseline comparison on the locked external validation set. Overall continuous-prediction performance is compared with training-derived median and mean inhibition-zone baselines. **Note:** Dashes indicate metrics that are not applicable to the corresponding analysis row.

Analysis	Model or measured IZ range	n	RMSE (mm)	MAE (mm)	R^2	Pearson r	Spearman ϱ	CCC	Mean measured IZ	Mean predicted IZ	Mean error (mm)	Median abs. error
Overall RegressionStack prediction	RegressionStack	1,130	4.48	3.20	0.618	0.787	0.792	0.758	13.69	13.85	0.16	2.37
Baseline comparison	TrainMedian_IZ_baseline	1,130	7.28	5.68	-0.009	—	—	—	—	—	-0.69	5.00
Baseline comparison	TrainMean_IZ_baseline	1,130	7.25	5.70	-0.000	—	—	—	—	—	-0.05	4.37

Abbreviations: CCC, concordance correlation coefficient; IZ, inhibition zone; MAE, mean absolute error; n, number of compounds; R^2, coefficient of determination; RMSE, root mean square error; ϱ, Spearman rank correlation coefficient.

Table S3 RegressionStack residual diagnostics stratified by measured inhibition-zone diameter on the locked external validation set. **Note:** Residuals were calculated as predicted IZ minus measured IZ; negative values indicate underprediction. Underpredicted percentage denotes the proportion of compounds with predicted IZ below the measured value. The active stratum includes compounds with measured IZ ≥25 mm and therefore overlaps with the 25–29.9 mm and ≥30 mm groups. Within-stratum R^2 values were omitted because restricted measured-IZ ranges can yield unstable or misleading estimates. Overall and baseline comparisons are provided in Table S2.

Measured-IZ stratum	*n*	MAE (mm)	RMSE (mm)	Mean residual (mm)	Median absolute error (mm)	Underpredicted (%)	Pearson *r*	Spearman *ρ*
<15 mm	646	2.967	4.231	2.022	2.114	28.6	0.609	0.630
15–19.9 mm	267	2.600	3.247	−0.661	2.291	59.2	0.312	0.308
20–24.9 mm	130	3.088	4.045	−2.700	2.255	86.2	0.302	0.348
25–29.9 mm	57	5.543	6.665	−5.527	4.938	94.7	0.180	0.169
≥30 mm	30	9.458	11.028	−9.458	7.451	100.0	−0.098	−0.216
Active stratum (≥25 mm)	87	6.893	8.428	−6.883	5.853	96.6	0.265	0.326

Abbreviations: IZ, inhibition zone; MAE, mean absolute error; *n*, number of compounds; RMSE, root mean square error; *r*, Pearson correlation coefficient; *ρ*, Spearman rank correlation coefficient.

Table S4. Performance comparison of the RegressionStack specialist regressors and final stacked model on the locked external validation set. Continuous-prediction metrics, threshold-independent ranking metrics, early enrichment, and top-*k* active recovery are reported separately for each first-level specialist regressor and the final stacked model. This comparison evaluates the performance of the individual specialist models relative to the complete ensemble; it is not a component-removal ablation analysis.

Model	Feature view	RMSE (mm)	MAE (mm)	R^2	ROC-AUC	PR-AUC	EF@1%	Top-25 hits	Top-50 hits	Top-100 hits
XGBoost_desc	desc	5.02	3.72	0.520	0.872	0.441	8.66	18	29	40
RF_morgan	morgan	4.76	3.46	0.568	0.881	0.471	8.66	18	28	45
GBM_morgan	morgan	5.14	3.75	0.497	0.858	0.463	10.82	19	31	39
XGBoost_maccs	maccs	4.67	3.38	0.585	0.905	0.549	9.74	18	33	52
ElasticNet_desc	desc	12.30	5.43	-1.883	0.719	0.177	2.16	6	15	24
RegressionStack	stacked_meta_regressor	4.48	3.20	0.618	0.896	0.545	9.74	19	33	49

Abbreviations: desc, descriptor-based feature view; EF@1%, enrichment factor at 1% of the ranked list; IZ, inhibition zone; MACCS, Molecular ACCess System; MAE, mean absolute error; PR-AUC, precision–recall area under the curve; R^2, coefficient of determination; RF, random forest; RMSE, root mean square error; ROC-AUC, receiver operating characteristic area under the curve; XGBoost, extreme gradient boosting.

Table S5. Top-k hit-list recovery and enrichment. Active recovery, PPV-k, and enrichment over random expectation are reported at k = 25, 50, and 100 for the locked external validation set and ChEMBL-derived transfer set.

Dataset	Model	k	Ranked compounds	Total actives	Prevalence	Random expected hits	Active hits	PPV-k	Enrichment vs random
Source external	SVC/MACCS	25	1,130	87	7.7%	1.9	16	0.640	8.31
		50	1,130	87	7.7%	3.8	31	0.620	8.05
		100	1,130	87	7.7%	7.7	49	0.490	6.36
	RegressionStack	25	1,130	87	7.7%	1.9	19	0.760	9.87
		50	1,130	87	7.7%	3.8	33	0.660	8.57
		100	1,130	87	7.7%	7.7	49	0.490	6.36
ChEMBL transfer	SVC/MACCS	25	1,458	75	5.1%	1.3	5	0.200	3.89
		50	1,458	75	5.1%	2.6	17	0.340	6.61
		100	1,458	75	5.1%	5.1	28	0.280	5.44
	RegressionStack	25	1,458	75	5.1%	1.3	12	0.480	9.33
		50	1,458	75	5.1%	2.6	19	0.380	7.39
		100	1,458	75	5.1%	5.1	32	0.320	6.22

Abbreviations: MACCS, Molecular ACCess System; PPV-k, positive predictive value at a fixed top-k list size; SVC, support vector classifier.

Table S6. Descriptor-reconstruction and prediction-agreement sensitivity analysis on the source-paper external validation set. Agreement between the original source-descriptor representation and the exact-name Mordred reconstruction was evaluated at the descriptor, prediction, ranking, and operating-threshold levels. **Note:** The 199 source descriptors were reconstructed by exact name using Mordred Community, with drift $>1 \times 10^{-6}$ in 32 columns. RegressionStack predictions from the reconstructed descriptors were compared with saved canonical predictions for the same 1,130 compounds. Therefore, the mean absolute predicted-IZ difference of 0.063 mm reflects prediction agreement, not MAE against measured IZ. As the descriptors were not reproduced exactly, these results were treated as a descriptor-reconstruction sensitivity analysis. Details of the 32 affected descriptors are provided in the companion drift-detail sheet.

Analysis level	Measure	Value	Unit
Descriptor agreement	Source external rows checked	1,130	rows
	Requested source-descriptor columns	199	columns
	Columns missing by exact name	0	columns
	Columns matching within 1×10^{-6}	167	columns
	Columns with numerical drift $>1 \times 10^{-6}$	32	columns
Prediction agreement	Mean absolute predicted-IZ difference	0.063	mm
	Median absolute predicted-IZ difference	3.844×10^{-7}	mm
	Maximum absolute predicted-IZ difference	1.736	mm
Ranking agreement	Saved canonical ROC-AUC	0.896	unitless
	Reconstructed ROC-AUC	0.896	unitless
	ROC-AUC change, reconstructed − saved	<0.001	unitless
	Saved canonical PR-AUC	0.545	unitless
	Reconstructed PR-AUC	0.554	unitless
	PR-AUC change, reconstructed − saved	0.010	unitless

Analysis level	Measure	Value	Unit
Threshold agreement	Saved PPV at predicted IZ ≥23.50 mm	0.689	unitless
	Reconstructed PPV at predicted IZ ≥23.50 mm	0.721	unitless
	Saved balanced accuracy at predicted IZ ≥23.50 mm	0.671	unitless
	Reconstructed balanced accuracy at predicted IZ ≥23.50 mm	0.672	unitless

Abbreviations: IZ, inhibition zone; PPV, positive predictive value; PR-AUC, precision–recall area under the curve; ROC-AUC, receiver operating characteristic area under the curve.

Table S7. Conventional out-of-fold and repeated scaffold-grouped validation performance for SVC/MACCS and RegressionStack. Conventional out-of-fold performance is compared with three repeated five-fold scaffold-grouped validation runs. **Note:** Results are from three repeated five-fold StratifiedGroupKFold partitions using Bemis–Murcko scaffolds and seeds 42, 101, and 202. Means and sample SDs were calculated across repeats, with scaffold groups kept exclusive to training or held-out folds. RegressionStack and SVC/MACCS retained the manuscript hyperparameters; RegressionStack models were retrained within each fold without repeating hyperparameter optimization. Active and high-IZ strata were defined as measured IZ ≥25 mm and ≥30 mm, respectively.

Model	Metric	Unit	Favorable direction	Conventional OOF	Repeat 1, seed 42	Repeat 2, seed 101	Repeat 3, seed 202	Scaffold-grouped mean	Scaffold-grouped SD
SVC/MACCS	ROC-AUC	Unitless	Higher	0.825	0.750	0.743	0.731	0.741	0.009
	PR-AUC	Unitless	Higher	0.393	0.292	0.290	0.281	0.288	0.006
	PPV	Unitless	Higher	0.519	0.407	0.374	0.395	0.392	0.016
	Balanced accuracy	Unitless	Higher	0.662	0.588	0.632	0.590	0.603	0.025
	EF@1%	Fold enrichment	Higher	8.146	7.332	6.110	6.110	6.517	0.706
RegressionStack	ROC-AUC	Unitless	Higher	0.860	0.796	0.786	0.770	0.784	0.013
	PR-AUC	Unitless	Higher	0.408	0.316	0.302	0.334	0.318	0.016
	PPV	Unitless	Higher	0.552	0.538	0.521	0.526	0.528	0.009
	EF@1%	Fold enrichment	Higher	8.961	8.961	6.924	8.961	8.282	1.176
	Overall MAE	mm	Lower	3.514	4.303	4.414	4.378	4.365	0.057
	Overall RMSE	mm	Lower	5.007	5.812	5.977	5.936	5.908	0.086
	R^2	Unitless	Higher	0.560	0.408	0.374	0.382	0.388	0.018

Model	Metric	Unit	Favorable direction	Conventional OOF	Repeat 1, seed 42	Repeat 2, seed 101	Repeat 3, seed 202	Scaffold-grouped mean	Scaffold-grouped SD
	Active-stratum MAE	mm	Lower	8.555	11.079	11.118	11.289	11.162	0.111
	High-IZ MAE	mm	Lower	11.150	14.189	13.988	14.179	14.119	0.113

Abbreviations: EF@1%, enrichment factor at 1% of the ranked list; IZ, inhibition zone; MACCS, Molecular ACCess System structural keys; MAE, mean absolute error; OOF, out-of-fold; PPV, positive predictive value; PR-AUC, precision–recall area under the curve; R^2, coefficient of determination; RMSE, root mean square error; ROC-AUC, receiver operating characteristic area under the curve; SD, standard deviation; SVC, support vector classifier.

Table S8. Scaffold-stratified external validation for SVC/MACCS and RegressionStack. PPV, sensitivity, balanced accuracy, ROC-AUC, and PR-AUC are reported separately for scaffold-shared compounds and scaffold-novel compounds.

Subset	Model	n	Actives	Prevalence	Unique scaffolds	Threshold	Pred. positives	TP	FP	PPV	Sensitivity	Balanced accuracy	ROC-AUC	PR-AUC
Scaffold-shared	SVC/MACCS	828	63	7.6%	433	0.360	52	30	22	0.577	0.476	0.724	0.877	0.496
Scaffold-novel	SVC/MACCS	302	24	7.9%	268	0.360	11	9	2	0.818	0.375	0.684	0.775	0.482
Scaffold-shared	RegressionStack	828	63	7.6%	433	23.50	36	23	13	0.639	0.365	0.674	0.907	0.541
Scaffold-novel	RegressionStack	302	24	7.9%	268	23.50	9	8	1	0.889	0.333	0.665	0.865	0.554

Abbreviations: FP, false positive; MACCS, Molecular ACCess System; PPV, positive predictive value; PR-AUC, precision–recall area under the curve; ROC-AUC, receiver operating characteristic area under the curve; SVC, support vector classifier; TP, true positive.

Table S9. Applicability-domain analysis. Predicted-positive counts and domain-stratified performance are reported for compounds inside and outside the Morgan/Tanimoto-defined applicability domain. The applicability-domain cutoff was a maximum training-set Tanimoto similarity of 0.474.

Model	Domain	n	Actives	Predicted positives	TP	FP	PPV	Mean max Tanimoto	Interpretation
SVC/MACCS	Inside AD	1,095	86	63	39	24	0.619	0.764	Interpretable; predicted positives present.
	Outside AD	35	1	0	0	0	—	0.274	PPV not estimable; no predicted positives.
RegressionStack	Inside AD	1,095	86	45	31	14	0.689	0.764	Interpretable; predicted positives present.
	Outside AD	35	1	0	0	0	—	0.274	PPV not estimable; no predicted positives.

Abbreviations: AD, applicability domain; FP, false positive; MACCS, Molecular ACCess System; PPV, positive predictive value; SVC, support vector classifier; Tanimoto, Tanimoto similarity coefficient; TP, true positive.

Table S10. SHAP-ranked MACCS feature attribution for the SVC/MACCS classifier. The top MACCS keys ranked by mean absolute SHAP value are shown with corresponding SMARTS definitions and structural interpretations. Feature attributions are model-specific associations and should not be interpreted as causal antibacterial mechanisms. **Note:** SMARTS patterns are RDKit/MACCS substructure definitions used for computational feature matching. Reader-facing structural summaries are approximate and should not be interpreted as causal antibacterial mechanisms.

Feature	MACCS index	Mean |SHAP|	RDKit SMARTS	Reader-facing structural summary
maccs_79	79	0.338	[#7]~*~*~[#7]	two nitrogen atoms separated through two intervening atoms (N~*~*~N)
maccs_57	57	0.207	[#8R]	oxygen atom in a ring
maccs_118	118	0.173	[$(*~[CH2]~[CH2]~*),$(*1~[CH2]~[CH2]1)]	two adjacent methylene groups or small ring CH2-CH2 pattern
maccs_42	42	0.169	F	contains fluorine
maccs_78	78	0.167	[#6]=[#7]	carbon-nitrogen double bond / imine-like C=N motif
maccs_125	125	0.156	?	RDKit MACCS key with no public SMARTS pattern; do not over-interpret
maccs_85	85	0.143	[#6]~[#7](~[#6])~[#6]	tertiary amine-like carbon-nitrogen-carbon branching
maccs_111	111	0.140	[#7]~*~[CH2]~*	nitrogen connected through one atom to methylene
maccs_153	153	0.138	[!#6;!#1]~[CH2]~*	heteroatom attached to methylene-substituted atom
maccs_69	69	0.134	[!#6;!#1]~[!#6;!#1;!H0]	heteroatom-heteroatom pattern involving at least one hydrogen-bearing heteroatom
maccs_82	82	0.130	*~[CH2]~[!#6;!#1;!H0]	Defined MACCS SMARTS pattern; no concise structural class assigned.
maccs_59	59	0.130	[#16]!:*:*	Defined MACCS SMARTS pattern; no concise structural class assigned.
maccs_133	133	0.122	*@*!@[#7]	Defined MACCS SMARTS pattern; no concise structural class assigned.

Feature	MACCS index	Mean ∣SHAP∣	RDKit SMARTS	Reader-facing structural summary
maccs_141	141	0.121	[CH3]	Defined MACCS SMARTS pattern; no concise structural class assigned.
maccs_146	146	0.121	[#8]	Defined MACCS SMARTS pattern; no concise structural class assigned.
maccs_104	104	0.113	[!#6;!#1;!H0]~*~[CH2]~*	Defined MACCS SMARTS pattern; no concise structural class assigned.
maccs_113	113	0.111	[#8]!:*:*	Defined MACCS SMARTS pattern; no concise structural class assigned.
maccs_53	53	0.111	[!#6;!#1;!H0]~*~*~*~[!#6;!#1;!H0]	Defined MACCS SMARTS pattern; no concise structural class assigned.
maccs_109	109	0.110	*~[CH2]~[#8]	Defined MACCS SMARTS pattern; no concise structural class assigned.
maccs_41	41	0.109	[#6]#[#7]	Defined MACCS SMARTS pattern; no concise structural class assigned.

Abbreviations: MACCS, Molecular ACCess System; RDKit, open-source cheminformatics toolkit; SHAP, SHapley Additive exPlanations; SMARTS, SMiles ARbitrary Target Specification.

Table S11. Nomination-overlap and operational complementarity summary. Score concordance, threshold-based nomination overlap, and top-25 active-hit overlap are summarized for SVC/MACCS and RegressionStack on the locked external validation set. **Note:** Predefined thresholds were SVC/MACCS probability ≥ 0.360 and RegressionStack predicted IZ ≥ 23.50 mm. Dashes indicate metrics that are not applicable to the corresponding row. Score-concordance rows report correlation values; top-25 rows report active-hit counts.

Analysis	Metric or nomination set	Selected compounds	TP	FP	PPV	Sensitivity	Value / active hits
Score concordance	Pearson correlation	—	—	—	—	—	0.523
	Spearman rank correlation	—	—	—	—	—	0.315
Threshold overlap	SVC/MACCS selected	63	39	24	0.619	0.448	—
	RegressionStack selected	45	31	14	0.689	0.356	—
	Intersection	35	27	8	0.771	0.310	—
	Union	73	43	30	0.589	0.494	—
Top-25 overlap	SVC/MACCS top-25 active hits	—	—	—	—	—	16
	RegressionStack top-25 active hits	—	—	—	—	—	19
	Shared top-25 active hits	—	—	—	—	—	9
	SVC/MACCS-only top-25 active hits	—	—	—	—	—	7
	RegressionStack-only top-25 active hits	—	—	—	—	—	10

Abbreviations: FP, false positive; IZ, inhibition zone; MACCS, Molecular ACCess System; PPV, positive predictive value; SVC, support vector classifier; TP, true positive.

Table S12. Tiered nomination, label-permutation null, and model-specific threshold-margin summary on the locked external validation set. Nomination composition, fixed-label permutation enrichment, and threshold-margin characteristics are summarized for the predefined model-selection tiers. **Note:** Near-boundary status was assigned when a model-specific positive margin satisfied either the absolute cutoff—0 to <0.05 probability units for SVC/MACCS or 0 to <1.0 mm for RegressionStack—or the corresponding relative first-quartile cutoff of 0.0587599 probability units for SVC/MACCS or 0.670473 mm for RegressionStack. All remaining model-specific nominations were classified as higher-margin. Permutation-null means represent the Monte Carlo averages across the 10,000 label permutations. Fold enrichment was calculated relative to the analytical random expectation based on nomination-set size and overall active prevalence.

Nomination tier	Compounds	Measured actives	Measured inactives	Active fraction	Sensitivity contribution	Permutation-null mean actives	Fold enrichment	Empirical p-value	Near-boundary count	Higher-margin count	Margin basis
Consensus-positive	35	27	8	0.771	0.310	2.69	10.02	0.0001	7 (SVC); 10 (RegressionStack)	28 (SVC); 25 (RegressionStack)	Both model-specific margins
SVC/MACCS-only	28	12	16	0.429	0.138	2.14	5.57	0.0001	9	19	SVC probability margin
RegressionStack-only	10	4	6	0.400	0.046	0.77	5.20	0.0055	5	5	RegressionStack predicted-IZ margin
Union	73	43	30	0.589	0.494	5.61	7.65	0.0001	See component tiers	See component tiers	Mixed model-specific margins
Both-negative	1,057	44	1,013	0.042	0.506	81.39	0.54	1.0000	Not applicable	Not applicable	Not applicable

Abbreviations: IZ, inhibition zone; MACCS, Molecular ACCess System structural keys; SVC, support vector classifier.

Table S13. Software versions and computational environment used for the primary analyses, descriptor reconstruction, and reviewer-requested sensitivity analyses.

Scope	Component	Version
Primary and descriptor-reconstruction environment	Operating system	macOS 26.3.1; arm64
	Python	3.12.13
	RDKit	2026.3.1
	Mordred Community	2.0.7
	scikit-learn	1.8.0
	XGBoost	3.2.0
	NumPy	2.4.4
	pandas	3.0.2
	SciPy	1.17.1
	SHAP	0.51.0
	Matplotlib	3.10.9
Source benchmark	Original 199 source descriptors	Generation version not reported
Reviewer sensitivity analyses	Python	3.9.6
	RDKit	2022.09.5
	scikit-learn	1.6.1
	XGBoost	2.1.4
	NumPy	1.26.4

Scope	Component	Version
	pandas	2.3.3
	SciPy	1.13.1
	Matplotlib	3.9.4
Randomization controls	Primary seed	42
	Scaffold-repeat seeds	42, 101, and 202

Abbreviations: MACCS, Molecular ACCess System structural keys; SHAP, SHapley Additive exPlanations; SVC, support vector classifier.

Table S14. Cross-validation and training-side model-selection design. Cross-validation settings used for out-of-fold prediction generation, training-side model selection, threshold selection, calibration, and RegressionStack meta-model construction are summarized. **Note:** Hyperparameter searches and subsequent OOF-prediction stages used only the training set, but hyperparameter selection was not nested within the OOF-generation folds. Accordingly, training OOF estimates are used for model and threshold construction and descriptive context rather than as unbiased generalization estimates. Final performance interpretation is based on the locked external validation set, which was not used for hyperparameter selection, calibration selection, meta-model fitting, or threshold selection.

Component	CV type	Folds	Shuffle	Seed	Stratification	Purpose
SVC/MACCS hyperparameter search	RandomizedSearchCV with StratifiedKFold	3	Yes	42	Binary activity label	Select SVC C and gamma by average precision using the full training set
SVC/MACCS calibrated OOF probability generation	Outer StratifiedKFold	5	Yes	42	Binary activity label	Generate calibrated OOF probabilities for threshold selection; each outer-training fit used internal five-fold isotonic calibration
SVC/MACCS final calibration and refit	CalibratedClassifierCV with StratifiedKFold	5	Yes	42	Binary activity label	Refit the selected SVC and isotonic calibrator on all training data before external prediction

RegressionStack specialist hyperparameter search	RandomizedSearchCV with KFold	5	Yes	42	None; continuous IZ target	Select specialist settings by negative MAE using the full training set
RegressionStack specialist OOF prediction	KFold	5	Yes	42	None; continuous IZ target	Generate specialist OOF predictions used as meta-regressor inputs
RegressionStack meta-regressor hyperparameter search	RandomizedSearchCV with KFold	5	Yes	42	None; continuous IZ target	Select XGBoost meta-regressor settings by negative MAE
RegressionStack meta-regressor OOF prediction	KFold	5	Yes	42	None; continuous IZ target	Generate OOF-predicted IZ values for training-side threshold selection

Abbreviations: CV, cross-validation; IZ, inhibition zone; MACCS, Molecular ACCess System; MAE, mean absolute error; OOF, out-of-fold; SVC, support vector classifier.

Table S15. Hyperparameter configurations. Final model components and hyperparameter settings used for SVC/MACCS, RegressionStack specialist regressors, the meta-regressor, and calibration are listed for reproducibility.

Model component	Parameter	Value
SVC/MACCS	method	isotonic
	cv	StratifiedKFold(n_splits=5, random_state=42, shuffle=True)
	estimator__scale__with_mean	True
	estimator__scale__with_std	True
	estimator__clf__kernel	rbf
	estimator__clf__C	5.6921660301037384
	estimator__clf__gamma	0.01638399383528232
	estimator__clf__class_weight	balanced
	estimator__clf__probability	True
	estimator__clf__random_state	42
	threshold	0.36
RegressionStack	effective_predicted_IZ_threshold	23.5
XGBoost_desc	n_estimators	390
	max_depth	7
	learning_rate	0.04652296584978102
	subsample	0.9369139098379994
	colsample_bytree	0.5247649977209133
	random_state	42
	n_jobs	-1
	objective	reg:squarederror
RF_morgan	n_estimators	402
	max_depth	None
	min_samples_leaf	1
	max_features	sqrt

Model component	Parameter	Value
	random_state	42
	n_jobs	-1
GBM_morgan	n_estimators	391
	max_depth	4
	learning_rate	0.12700582421772894
	subsample	0.996884623716487
	random_state	42
XGBoost_maccs	n_estimators	351
	max_depth	6
	learning_rate	0.10440040750544663
	colsample_bytree	0.5290418060840998
	random_state	42
	n_jobs	-1
	objective	reg:squarederror
ElasticNet_desc	alpha	0.01474275315991467
	l1_ratio	0.4297256589643226
	max_iter	5000
	random_state	42
RegressionStack_meta_XGBoostRegressor	n_estimators	200
	max_depth	2
	learning_rate	0.05
	subsample	0.9
	random_state	42
	n_jobs	-1
	objective	reg:squarederror

Abbreviations: GBM, gradient boosting machine; IZ, inhibition zone; MACCS, Molecular ACCess System; RF, random forest; SVC, support vector classifier; XGBoost, extreme gradient boosting.
